# Interplay between *AHR* genotypes, lifestyle factors and adjuvant breast cancer treatments significantly impacts clinical outcome in a population-based cohort

**DOI:** 10.1038/s44276-025-00167-w

**Published:** 2025-07-11

**Authors:** Annelie Augustinsson, Christopher Godina, Linn Nilsson, Kelin Gonçalves de Oliveira, Karolin Isaksson, Helena Jernström

**Affiliations:** 1 Department of Clinical Sciences in Lund, Oncology, Lund University and Lund University and Lund University Cancer Center LUCC/Kamprad, Lund, Sweden; 2 Skåne University Hospital, Lund, Sweden; 3 Department of Medical Physics and Engineering, Växjö Central Hospital and Department of Research and Development, Region Kronoberg, Växjö, Sweden; 4 Department of Clinical Sciences in Lund, Surgery, Lund University and Lund University Cancer Center LUCC, Lund, Sweden; 5 Kristianstad Hospital, Kristianstad, Sweden

## Abstract

**Background:**

The purpose was to evaluate the prognostic impact of aryl hydrocarbon receptor (*AHR*) genotypes in relation to lifestyle and adjuvant treatments in breast cancer.

**Methods:**

*AHR* genotyping was performed on genomic DNA from 1701 patients included 2002–2016 in Lund, Sweden, and followed for up to 15 years. Eight *AHR* polymorphisms and eight haplotypes were analysed using survival and interaction analyses in relation to prognosis.

**Results:**

Homozygosity for major allele frequencies was 42.1%–88.5%. *AHR* genotypes linked to lower *AHR* expression conferred differential prognosis combined with smoking, alcohol, antioxidant supplements, chemotherapy or endocrine therapy, with interactions between exposures and genotypes. Preoperative antioxidants combined with minor alleles of *AHR*_6 or *AHR*_9 conferred three-fold risks for breast cancer events, not seen in other patients (*P*_interactions_ ≤ 0.016). Interactions between the CGATTAGC haplotype and chemotherapy revealed five-fold risks for breast cancer events or death compared to other haplotypes or no chemotherapy (*P*_interactions_ ≤ 0.010). *AHR* genotypes were not prognostic in radiation therapy-treated patients.

**Conclusions:**

The prognostic impact of *AHR* genotypes depended on lifestyle and treatments, possibly due to the role of AhR as master regulator of metabolism, hypoxia, DNA repair and immune response. If confirmed, these findings may contribute to more personalised lifestyle recommendations and treatment.

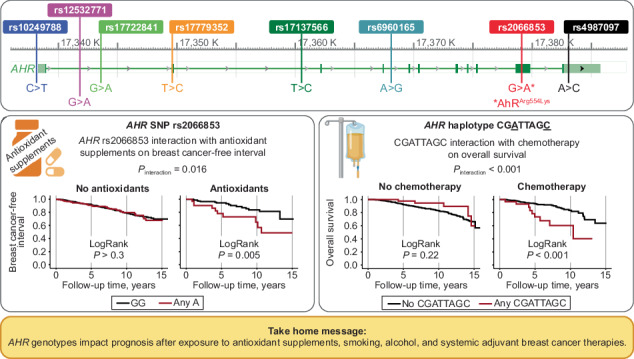

## Background

Breast cancer accounts for a substantial proportion of cancer related morbidity and mortality in women [1, 2]. Breast cancer is a heterogeneous disease. Despite new therapeutic modalities, the prognosis remains poor in some subgroups [3]. Identification of novel prognostic and predictive biomarkers is crucial. In addition to tumour characteristics, inter-individual differences in prognosis and medication response depend on host factors, including lifestyle and genetic variation. Multiple gene products affect both pharmacokinetics and pharmacodynamics [4]. The multifunctional transcription factor aryl hydrocarbon receptor (AhR) merits further investigation as biomarker in the pharmacogenomic setting.

AhR is encoded by the *AHR* gene, comprising 11 exons located on chromosome 7 [5]. *AHR* is highly polymorphic. Previous studies have reported associations between *AHR* single nucleotide polymorphisms (SNPs) and breast cancer risk [6–8]. AhR is activated upon exposure to a wide range of ligands, including toxins (*e.g*. dioxin), prescription drugs (*e.g*. raloxifene and 4-hydroxytamoxifen), tobacco smoke, cruciferous vegetables and tryptophan metabolites [9–12]. Inactivate AhR is localised in the cytoplasm. Ligand binding results in translocation into the nucleus, where AhR forms a complex with the AhR nuclear translocator (ARNT) and stimulates transcription of target genes. These include cytochrome P450 (CYP) genes, such as *CYP1A1*, *CYP1A2, CYP1B1* and *CYP19A1* (aromatase), encoding enzymes that contribute to carcinogenesis and hormone metabolism [13]. Aromatase converts androgens into oestrogens [14] and is targeted by aromatase inhibitors (AIs). AhR ligands have anti-oestrogen effects through competitive binding of the oestrogen receptor (ER) to oestrogen response elements, thereby suppressing ER-regulated transcription [15]. In patients with ER+ breast cancer, the prognostic impact of AhR is context dependent [16–18].

A review reported that AhR acts as a negative regulator of anti-tumour immunity by promoting AhR driven cancer cell motility and suppressing the adaptive immunity [19]. AhR also regulates the inflammatory process through pro-inflammatory cytokines, *e.g*. interleukin 6 (IL-6) and interleukin 8 (IL-8), and the maturation and activity of immune cells. Furthermore, AhR interacts with coordinators of inflammatory response and breast cancer inducing factors such as nuclear factor kappa-light-chain-enhancer of activated B cells (NF-κB). Hence, AhR activation in breast tumour tissue promotes a chronic inflammatory state, especially in more aggressive subtypes, including triple-negative breast cancer (TNBC) [19]. A recent study reported that AhR activity suppresses STImulator of Interferon Genes (STING) mediated type I interferon (IFN-I) expression in TNBC cell lines and that AhR could potentially represent a feasible therapeutic strategy to enhance the immunogenicity of TNBC tumours [20].

The AhR ligands function as selective AhR modulators (SAhRMs) [21], exhibiting agonistic or antagonistic effects on several hallmarks of cancer, *e.g*. evading growth suppressors, sustained proliferative signals, resistance to cell death, inflammation and activation of invasion and metastasis [22]. A clinical phase I trial studying an AhR inhibitor in patients with advanced small cell lung cancer or head and neck cancer has recently been completed (NCT04069026), highlighting that AhR merits further investigation in the cancer setting.

We hypothesise that different *AHR* genotypes may differentially impact breast cancer prognosis depending on exposures to AhR ligands. The purpose was to perform a comprehensive analysis of the prognostic impact of *AHR* genotypes, lifestyle and different adjuvant treatments in patients with primary breast cancer.

## Methods

### Study population

**Fig. 1:**
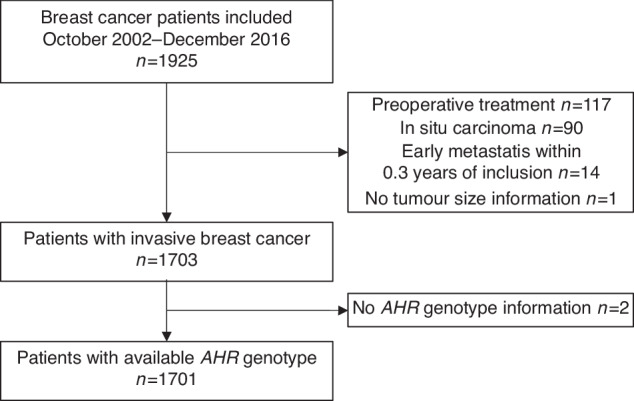
Flowchart. Flowchart of included and excluded breast cancer patients.

The Breast cancer and blood (BCblood) study is a population-based prospective cohort consisting of patients with a first primary breast cancer from Skåne University Hospital, Lund, Sweden. The Ethical Review Board in Lund approved the study (Dnr 75–02, 37–08, 658–09, and amendments). Patients were included between diagnosis and surgery. Patients who had been diagnosed with any type of cancer within ten years were not included. Between October 2002 and December 2016, 1925 breast cancer patients were included. All participants provided written informed consent. Patients who had preoperative treatment (*n* = 117), *in situ* carcinoma only (*n* = 90), early recurrence within 0.3 years (*n* = 14), tumour size missing (*n* = 1) or genotype missing (*n* = 2) were excluded (Fig. 1).

Previous studies have described the BCblood cohort in detail [23, 24]. To summarize, patients completed a three-page questionnaire preoperatively, including information on reproductive factors, smoking, alcohol and medication intake. Preoperative antioxidant supplements included vitamin A, C, E, carotenoids or Q10 [25, 26]. Blood samples and body measurements were collected by research nurses preoperatively, and body mass index (BMI) was calculated. The cut-off for overweight was set to ≥25 kg/m^2^ [27]. Clinicopathological information was collected from patient charts and pathology reports. ER and progesterone receptor (PgR) positivity cut-offs were 10% stained nuclei, as per Swedish clinical guidelines [28]. HER2 evaluation was not incorporated into clinical practice until November 2005. For patients included 2002–2012 with missing human epidermal growth factor receptor 2 (HER2) status, the HER2 status (amplified/non-amplified) was obtained from dual gene protein staining of HER2 on tumour tissue arrays (TMAs), with 97.7% agreement with available pathological assessments [29]. Tumour-specific AhR staining was obtained from TMAs and dichotomized into positive/negative for malignant cells and ‘strong’/’not strong’ for stromal cells [18].

Patients were followed until 30 June 2019. Information regarding breast cancer events (including locoregional recurrence, contralateral breast cancer and distant metastasis) and date of death was obtained from patient charts and the Population Registry.

### Genotyping

DNA from leukocytes in the blood samples was extracted using DNeasy® Blood and Tissue kit and processed with QiaCube (Qiagen, Hilden, Germany) according to the manufacturers’ instructions. SNP genotyping was performed using OncoArray by Illumina [30] at the Centre for Translational Genomics at Lund University. The OncoArray platform has a genome-wide backbone, comprising 230,000 SNPs tagging most common genetic variants. In addition, the OncoArray contains pharmacogenetic markers, densely mapped SNPs of known susceptibility regions, rare variants from sequencing experiments and cancer related traits. Standard quality control was performed on all scans. All samples with SNPs with a minor allele frequency <1% (*n* = 6), or a call rate <95% (*n* = 1) were excluded. Eight out of fifteen *AHR* SNPs, rs10249788 (*AHR*_1), rs12532771 (*AHR*_3), rs17722841 (*AHR*_4), rs17779352 (*AHR*_5), rs17137566 (*AHR*_6), rs6960165 (*AHR*_7), rs2066853 (*AHR*_9) and rs4987097 (*AHR*_11), passed quality control (Supplementary Table 1).

### *AHR* haplotype construction

**Fig. 2:**
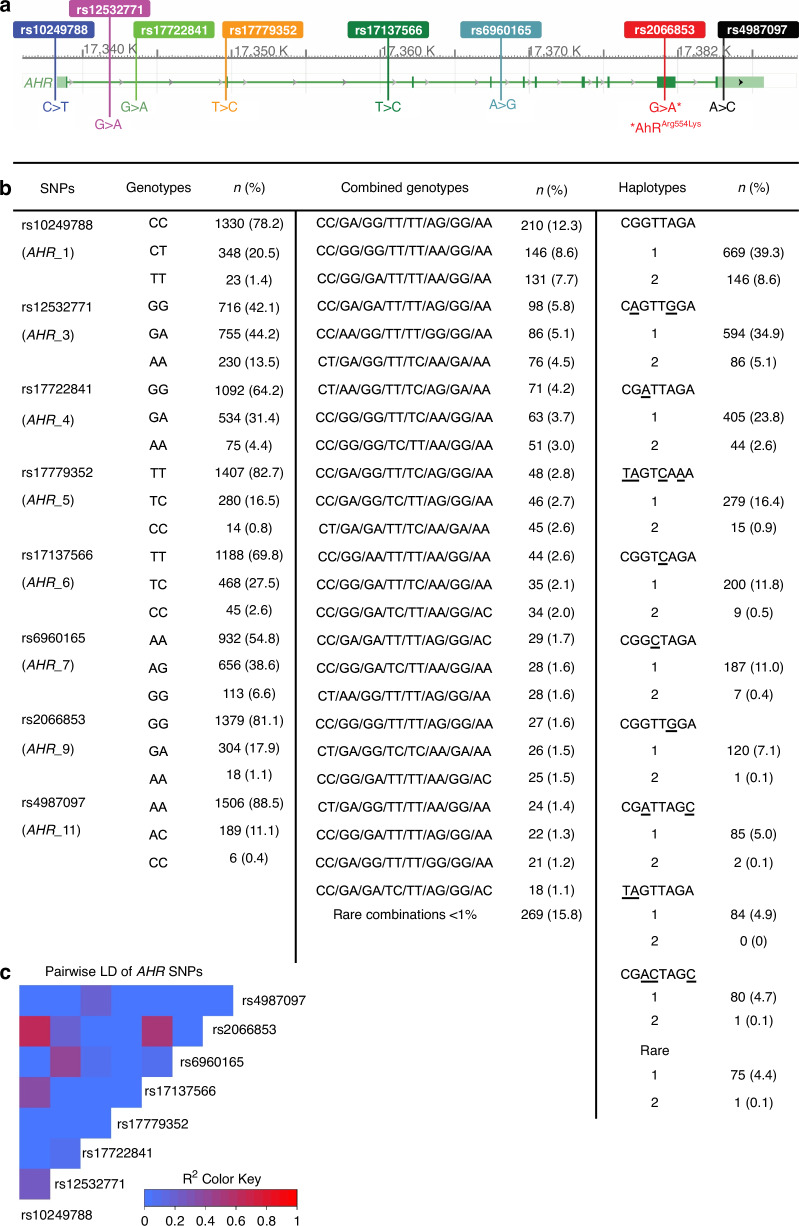
*AHR* genotypes, genomic region, frequencies and linkage. **a** The genomic region of *AHR*. **b** Frequencies of *AHR* SNPs, combined genotypes and haplotypes for the 1701 breast cancer patients. **c** A linkage disequilibrium heatmap of the linkage between the *AHR* SNPs.

The major allele for all eight SNPs were used as reference for all statistical analyses (Fig. 2a). Six SNPs (*AHR*_1, *AHR*_4, *AHR*_5, *AHR*_6, A*HR*_9 and *AHR*_11) were dichotomized into none (0) and any minor allele (1+) due to low frequencies (<50 patients) of homozygous minor allele carriers, while *AHR*_3 and *AHR*_7 were analysed in three groups each (Fig. 2b). Each SNP was cross-tabulated against the other seven SNPs, and based on the most likely combinations haplotypes and combined genotypes were constructed. The SNPs for *AHR*_6 and *AHR*_11 were missing for nine and 23 patients, respectively, and the most likely alleles were imputed based on the other genotypes. The haplotypes were compared to a reference European population (1000Genomes) [5] (Supplementary Table 2). The two most common haplotypes, CGGTTAGA, with no SNP variants, and CAGTTGGA, with SNP variants on *AHR*_3 and *AHR*_7, were analysed in three groups each. Conversely, the six haplotypes CGATTAGA, TAGTCAAA, CGGTCAGA, CGGCTAGA, CGGTTGGA and CGATTAGC were dichotomized into none (0) and any carrier (1+) due to low frequencies (<5%) of homozygous haplotype carriers (Fig. 2b).

Database searches for proxy and putatively functional variants and expression quantitative trait loci (eQTL) in linkage disequilibrium with the eight SNPs were performed using LDLinkR [31] in R (v4.0.2). LDheatmap was used to generate a linkage disequilibrium heatmap (Fig. 2c).

### Differential gene expression analysis

Data from 5326 patients with clinical follow-up in the Swedish Cancerome Analysis Network–Breast (SCAN-B) was used to perform bioinformatics analysis, as described previously [32, 33]. A differential gene expression (DGE) analysis to find differentially expressed genes (DEGs) between *AHR*-high (tertile three (T3) of *AHR* gene expression) and *AHR*-low (tertile one (T1) of *AHR* gene expression) tumours was performed using the Limma-Voom package [34]. The criteria used to define DEGs was a false discovery rate (FDR) of ≤0.05 and log_2_ fold change (log_2_FC)≥1.5 for upregulated genes and log_2_FC ≤ −1.5 for downregulated genes. To find concordant gene sets that differed between *AHR-*high and *AHR*-low samples, gene set enrichment analysis (GSEA) was performed in clusterProfiler [35] and gene sets were grouped according to hallmark signature annotations [36].

### Statistical analysis

Statistical analyses were performed using SPSS software (IBM SPSS Statistics for Windows, version 28.0, Armonk, NY, IBM Corp). Dichotomized variables included age (≥50 years), BMI (≥25 kg/m^2^), parous, ever use of menopausal hormone therapy (MHT), preoperative smoking, alcohol abstention, preoperative antioxidant use, invasive tumour size (>20 mm or skin or muscular involvement, *i.e*. pT2/3/4), any axillary lymph node involvement, histological grade III, ER+, PgR+, HER2 amplification, TNBC, AhR cytoplasmic (high), AhR nuclear positivity, and adjuvant chemotherapy, radiation therapy, trastuzumab, tamoxifen (TAM), AI or endocrine treatment in four groups: none, TAM only, AI only or sequential (TAM/AI or AI/TAM).

The eight individual SNPs and eight haplotypes were analysed in relation to patient and tumour characteristics using Pearson’s chi-square test. To estimate the fraction of patients without breast cancer events, Kaplan-Meier curves were generated, and statistical significance was calculated with the LogRank test. Cox regression models were used for multivariable analyses, yielding hazard ratios (HRs) with 95% confidence intervals (CIs). Adjustments were performed for age at inclusion (years), tumour characteristics (invasive tumour size, axillary node involvement and histological grade) and adjuvant treatments (chemotherapy, radiation therapy, TAM or AI). The main endpoint was any breast cancer event. Overall survival (death due to any cause) was used as secondary endpoint. Breast cancer-free interval was censored at last follow-up, prior to emigration or at death by 30 June 2019. Formal two-way interaction analyses were performed between *AHR* genotypes and lifestyle factors or adjuvant treatments in multivariable Cox regression models. The interaction analyses for endocrine treatments were restricted to patients with ER+ tumours.

Power calculations were performed using the PS Power and Sample Size Program, version 3.1.6 (Vanderbilt University, TN, USA) [37]. For the power calculation we assumed that with 1,700 patients and minor allele frequencies varying between 11.5% and 58.0%, a 14-year accrual time with an additional follow-up of three years, 80% probability (power), *α* of 0.05 and with a median time to event of 5.1 years, it would be possible to detect true HRs of ≤0.78 or ≥1.30 for 11.5% and ≤0.85 or ≥1.18 for 58.0%, respectively. All *P* values were two-tailed and a *P* value < 0.05 was considered statistically significant. Nominal *P* values are presented without adjustments for multiple testing due to the exploratory nature of the study [38].

## Results

### *AHR* SNPS in relation to *AHR* mRNA levels

First, eQTL analyses of the potential impact of the eight SNPs on *AHR* mRNA expression were performed. For four of the SNPs (*AHR*_1, *AHR*_3, *AHR*_6 and *AHR*_9), the minor allele was either directly associated with or linked to other genetic variants in *AHR* that were associated with lower *AHR* mRNA expression in whole blood. *AHR*_9 is functional (AhR^Arg554Lys^). In contrast, the minor allele of *AHR*_4 was associated with higher *AHR* mRNA expression, while the minor allele of *AHR*_7 was associated with lower *AHR* mRNA expression, in testis (Supplementary Table 3).

### Clinicopathological data in relation to *AHR* genotypes

The median age at inclusion was 62.0 years (interquartile range (IQR), 52.1–69.3 years) for all 1701 patients. Descriptive statistics for all patients are presented in Table 1, and in relation to *AHR* genotypes in Supplementary Table 4. The homozygosity for the major allele frequencies ranged from 42.1% to 88.5%. There were few differences according to *AHR* genotypes. Patients with *AHR*_11 Any C were more often axillary node positive compared to patients with the *AHR*_11 AA genotype (*P* = 0.044). Regarding adjuvant breast cancer treatments, patients with the *AHR*_7 AA genotype were more often treated with trastuzumab compared with patients with *AHR*_7 AG or AHR7 GG (*P* = 0.044). No other statistically significant differences according to *AHR* genotype were observed, including tumour-specific cytoplasmic or nuclear AhR protein levels.

**Table 1:** Clinicopathological characteristics of included patients, *n* = 1701.

	Patients		Missing
	*n*	%	*n*
Age ≥ 50 years	1367	80.4	0
BMI ≥ 25 kg/m^2^	836	52.3	101
Parous	1509	88.7	0
Ever use of MHT	671	39.6	6
Preoperative smoker	300	17.7	7
Alcohol abstainer	198	11.7	7
Antioxidant supplement user	175	10.4	24
Invasive tumour size > 20 mm or skin/muscular involvement	441	25.9	0
Axillary node involvement ≥ 1	579	34.1	2
Histological grade III	470	27.7	6
Hormone receptor status			
ER positive	1504	88.5	2
PgR positive	1214	71.5	2
HER2 amplification^a^	150	10.7	11
Triple negative	129	7.6	8
AhR localisation^b^			
Cytoplasmic high	736	80.1	782
Nuclear positive	291	31.7	782
Treatment by last follow-up prior to any event			
Adjuvant chemotherapy	488	28.7	0
Adjuvant radiation therapy	1129	66.4	0
Adjuvant trastuzumab^a^	118	8.3	0
Patients with ER+ tumours, *n* = 1504			
Adjuvant TAM	892	59.3	0
Adjuvant AI	688	45.7	0
Adjuvant endocrine treatment			
No	322	21.4	0
TAM only	494	32.8	0
AI only	290	19.3	0
Sequential therapy (TAM/AI or AI/TAM)	398	26.5	0

*AhR* aryl hydrocarbon receptor, *AI* aromatase inhibitor, *BMI* body mass index, *ER* oestrogen receptor, *HER2* human epidermal growth factor receptor 2, *MHT* menopausal hormone therapy, *PgR*progesterone receptor, *TAM* tamoxifen.

^a^2005–2016, *n* = 1415.

^b^Adjusted for time between surgery and staining.

### *AHR* genotypes in relation to prognosis

The patients were followed for up to 15 years after inclusion. The median follow-up time for the 1333 patients still at risk was 5.1 years (IQR, 3.1–9.1 years). During follow-up, there were 238 patients with breast cancer events, including 74 with locoregional recurrence, 149 with distant metastasis and 59 with contralateral breast cancer. Additionally, 218 patients died during follow-up, of which 113 had a prior breast cancer event. None of the *AHR* SNPs were associated with breast cancer events (all *P* values ≥ 0.070) or overall survival (all *P* values ≥ 0.24).

The most likely haplotypes were constructed for each patient (Fig. 2b). The haplotype frequencies >5% in the BCblood cohort were in the same order as the expected haplotype frequencies in the European 1000Genome human population (Supplementary Table 2). The eight *AHR* haplotypes with frequencies >5% were analysed in relation to prognosis. Homozygosity for the most common haplotype, CGGTTAGA, was associated with a higher risk of breast cancer events, but only in the multivariable analysis (adjusted for age at inclusion, tumour characteristics and adjuvant treatments), HR_adj_ 1.56 (95% CI 1.02–2.39), compared to no copies. Conversely, having any copy of the haplotype CGGTCAGA was associated with reduced risk for breast cancer events in both the univariable (LogRank 2 df *P* = 0.024) and multivariable analyses, HR_adj_ 0.56 (95% CI 0.35-0.91). No associations between *AHR* haplotypes and overall survival were observed.

### Interactions between lifestyle factors and *AHR* genotypes on prognosis

**Fig. 3:**
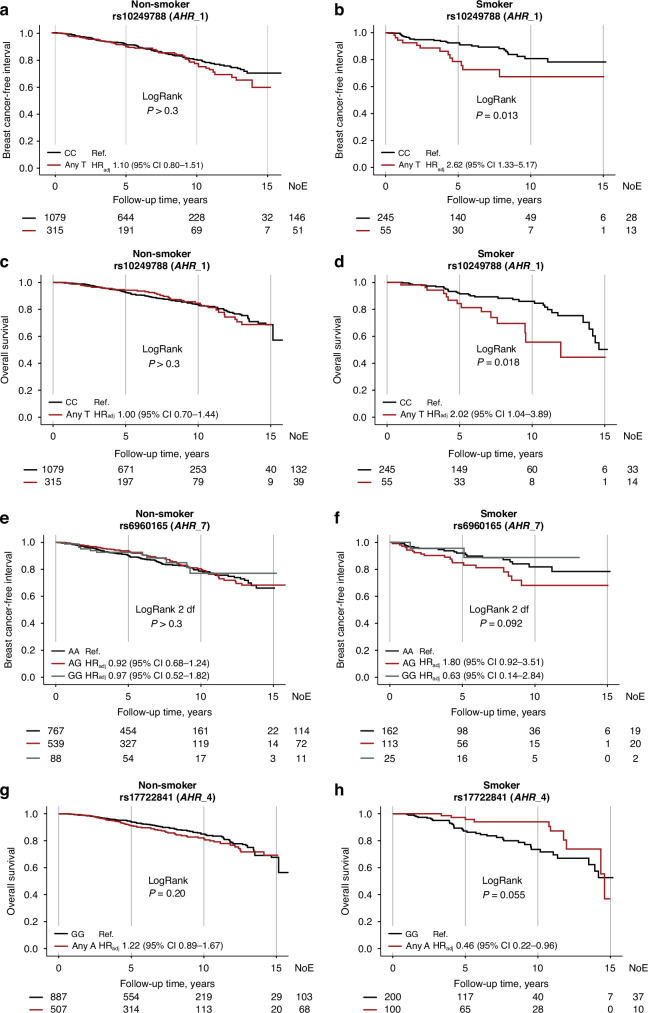
*AHR* genotypes, smoking and prognosis. Kaplan-Meier estimates of breast cancer-free interval in relation to *AHR*_1, with LogRank and HR_adj_ (95% CI), in **a** non-smokers and **b** smokers. Overall survival in relation to *AHR*_1, with LogRank and HR_adj_ (95% CI), in **c** non-smokers and **d** smokers. Overall survival in relation to *AHR*_4, with LogRank and HR_adj_ (95% CI), in **e** non-smokers and **f** smokers. Breast cancer-free interval in relation to *AHR*_7, with LogRank and HR_adj_ (95% CI), in **g** non-smokers and **h** smokers. The number of patients is indicated at each time-point. The study is ongoing. Thus, the number of patients decreases with time.

In order to study if there were interactions between lifestyle factors and different *AHR* genotypes, formal two-way interaction analyses were performed. No interactions were found between *AHR* SNPs or haplotypes and age ≥50 years or BMI ≥ 25 kg/m^2^ (all *P* values ≥ 0.10). Conversely, interactions were found between self-reported preoperative smoking and *AHR*_1 Any T on breast cancer events, HR_adj_ 2.41 (95% CI 1.15–5.02; *P*_interaction_ = 0.019), and overall survival, HR_adj_ 2.30 (95% CI 1.11–4.78; *P*_interaction_ = 0.026). Among smokers, two-fold risks for breast cancer events and death were seen in patients with *AHR*_1 Any T, using *AHR*_1 CC as reference, but not in non-smokers (Fig. 3a–d). There was also an interaction between smoking and the *AHR*_7 AG genotype on breast cancer events, HR_adj_ 2.12 (95% CI 1.05–4.26; *P*_interaction_ = 0.036), using *AHR*_7 AA as reference. However, no statistically significant associations were seen in the subgroup analysis after stratification according to smoking status (Fig. 3e, f). Additionally, an interaction was found between smoking and *AHR*_4 on overall survival, HR_adj_ 0.40 (95% CI 0.18–0.86; *P*_interaction_ = 0.018), where smokers with *AHR*_4 Any A had a better prognosis compared to *AHR*_4 GG. This was not seen among non-smokers (Fig. 3g, h).

There was no association between preoperative smoking and alcohol abstention. However, interactions were found between alcohol abstention and *AHR*_1 Any T on breast cancer events, HR_adj_ 0.33 (95% CI 0.13–0.84; *P*_interaction_ = 0.020), and overall survival, HR_adj_ 0.37 (95% CI 0.15–0.94; *P*_interaction_ = 0.035). Among patients who self-reported alcohol abstention, *AHR*_1 Any T carriers had a non-significant decreased risk for breast cancer events compared with *AHR*_1 CC carriers (Supplementary Fig. 1A). Moreover, patients with *AHR*_1 Any T who self-reported drinking alcohol had 44% higher risk for breast cancer events compared to *AHR*_1 CC carriers (Supplementary Fig. 1B). No statistically significant association between *AHR*_1 and overall survival was seen in the subgroup analyses after stratification according to alcohol use (Supplementary Fig. 1C, D). An interaction was also found between alcohol abstention and having one copy of the haplotype CAGTTGGA on overall survival, HR_adj_ 2.22 (95% CI 1.06–4.65; *P*_interaction_ = 0.034), but the subgroup analyses revealed no statistically significant associations (Supplementary Fig. 1E, F).

**Fig. 4:**
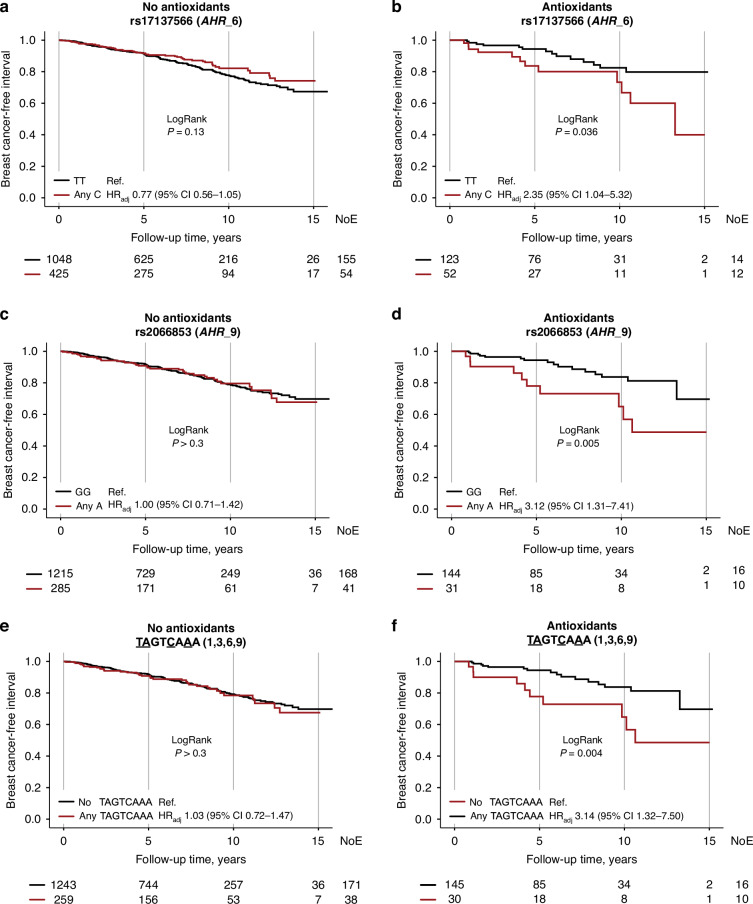
*AHR* genotypes, antioxidant supplement use and prognosis. Kaplan-Meier estimates of breast cancer-free interval, with LogRank and HR_adj_ (95% CI), in **a**, **c**, **e** non-users of antioxidants and **b**, **d**, **f** antioxidant users in relation to *AHR*_6, *AHR*_9 and TAGTCAAA genotypes, respectively. The number of patients is indicated at each time-point. The study is ongoing. Thus, the number of patients decreases with time.

With respect to self-reported preoperative intake of antioxidant supplements, interactions were found between antioxidant use and *AHR*_6 Any C, HR_adj_ 3.08 (95% CI 1.34–7.10; *P*_interaction_ = 0.008), and *AHR*_9 Any A, HR_adj_ 2.91 (95% CI 1.22–6.94; *P*_interaction_ = 0.016), on breast cancer events. Antioxidant users with *AHR*_6 Any C had a two-fold risk for breast cancer events compared to *AHR*_6 TT carriers, while non-antioxidant users with *AHR*_6 Any C had somewhat lower risk for breast cancer events compared to carriers of *AHR*_6 TT (Fig. 4a, b). Additionally, antioxidant users with *AHR*_9 Any A had a three-fold increased risk for breast cancer events, using *AHR*_9 GG as reference, but no association was seen among non-antioxidant users (Fig. 4c, d). There was also an interaction between any copy of the TAGTCAAA haplotype and antioxidant use, HR_adj_ 2.87 (95% CI 1.20–6.87; *P*_interaction_ = 0.018). Among antioxidant users, a three-fold increased risk for breast cancer events was seen in patients with the any TAGTCAAA genotype, but not in non-users (Fig. 4e, f). No interactions were seen between antioxidant use, *AHR* genotypes and overall survival. Taken together, smoking, alcohol and use of antioxidant supplements conferred poorer prognosis in patients carrying an *AHR* variant associated with lower *AHR* mRNA expression.

### Interactions between adjuvant breast cancer treatments and *AHR* genotypes on prognosis

**Fig. 5:**
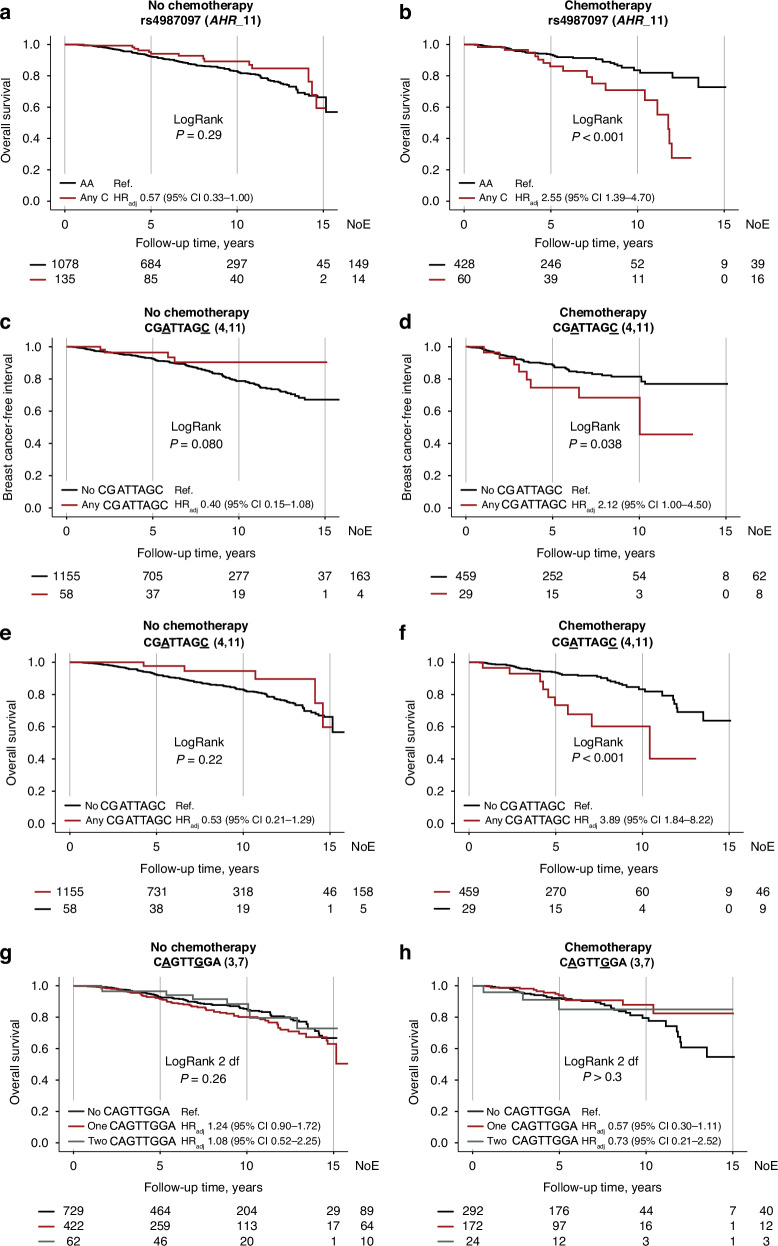
*AHR* genotypes, chemotherapy and prognosis. Kaplan-Meier estimates of overall survival in relation to *AHR*_11, with LogRank and HR_adj_ (95% CI), in **a** chemonaïve and **d** chemotherapy-treated patients. Breast cancer-free interval in relation to CGATTAGC genotype, with LogRank and HR_adj_ (95% CI), in **c** chemonaïve and **d** chemotherapy-treated patients. Overall survival in relation to CGATTAGC or CAGTTGGA genotypes, with LogRank and HR_adj_ (95% CI), in **e**, **g** chemonaïve and **f**, **h** chemotherapy-treated patients. The number of patients is indicated at each time-point. The study is ongoing. Thus, the number of patients decreases with time.

Formal interaction analyses were performed between adjuvant treatments and the different *AHR* genotypes. None of the *AHR* SNPs interacted with adjuvant chemotherapy regarding breast cancer events. However, for overall survival, a strong interaction was found between adjuvant chemotherapy and *AHR*_11 Any C, HR_adj_ 4.80 (95% CI 2.12–10.87; *P*_interaction_ < 0.001). Chemotherapy-treated patients with *AHR*_11 Any C had a two-fold increased risk for death, and the opposite was seen in chemonaïve patients, using *AHR*_11 AA as reference (Fig. 5a, b). Strong interactions were also found between chemotherapy and having any copy of CGATTAGC on breast cancer events, HR_adj_ 5.11 (95% CI 1.48–17.70; *P*_interaction_ = 0.010) and overall survival, HR_adj_ 8.22 (95% CI 2.59–26.15; *P*_interaction_ < 0.001). Among chemotherapy-treated patients, a two-fold increased risk for breast cancer events, and a three-fold risk for death, were seen in patients with any CGATTAGC. Conversely, in chemonaïve patients, somewhat decreased risks for breast cancer events and death were seen (Fig. 5c–f). There was also an interaction between chemotherapy and having one copy of CAGTTGGA, HR_adj_ 0.43 (95% CI 0.21–0.89; *P*_interaction_ = 0.022), but not two copies, HR_adj_ 0.66 (95% CI 0.16–2.67; *P*_interaction_ > 0.3), on overall survival, compared to having no copies. However, no statistically significant association was found in the subgroup analysis after stratification for chemotherapy (Fig. 5g, h). Taken together, chemotherapy conferred poorer prognosis in patients carrying any *AHR* variant associated with lower *AHR* mRNA expression.

No interactions were seen between radiation therapy and *AHR* SNPs on breast cancer events or overall survival. Interactions were found between radiation therapy and having one common CGGTTAGA haplotype copy, HR_adj_ 0.53 (95% CI 0.30–0.93; *P*_interactions_ = 0.027), or two copies, HR_adj_ 0.38 (95% CI 0.16–0.90; *P*_interactions_ = 0.027), on breast cancer events. Among radiation therapy-treated patients, there was no association between having one or two CGGTTAGA copies and breast cancer events. Conversely, homozygous carriers of CGGTTAGA, not treated with radiation therapy, had a two-fold higher risk for breast cancer events compared to non-carriers (Supplementary Fig. 2A, B). There was also an interaction between radiation therapy and having any copy of the CGGCTAGA haplotype, HR_adj_ 3.33 (95% CI 1.25–8.86; *P*_interaction_ = 0.016). There was no difference in breast cancer events between radiation therapy-treated patients with or without the CGGCTAGA haplotype. Conversely, patients with any copy of CGGCTAGA who had not received radiation therapy had decreased risk for breast cancer events, compared with non-carriers of CGGCTAGA (Supplementary Fig. 2C, D). Thus, any differences between *AHR* genotypes were only observed in non-radiation therapy-treated patients. The effect sizes remained essentially the same after further adjustment for mastectomy as final type of surgery (*n* = 647). No interactions between *AHR* haplotypes and radiation therapy on overall survival were found. Taken together, *AHR* variants were only associated with prognosis in non-radiotherapy-treated patients.

Only patients with ER+ tumours were included in the interaction analyses between *AHR* genotypes and adjuvant endocrine treatments. Interactions were found between the use of AIs and *AHR*_3 GA on breast cancer events, HR_adj_ 1.92 (95% CI 1.03–3.57; *P*_interaction_ = 0.040) and overall survival, HR_adj_ 2.07 (95% CI 1.08–3.97; *P*_interaction_ = 0.029), using *AHR*_3 GG as reference. Among AI-treated patients, higher risks for breast cancer events and death were seen in *AHR*_3 GA carriers. This was not seen in non-AI-treated patients (Supplementary Fig. 3A–D). No interactions were seen between *AHR* haplotypes and AI treatment on prognosis.

No interactions were found between *AHR* genotypes and TAM treatment on breast cancer events. However, an interaction on overall survival was seen between the *AHR*_7 AG genotype and use of TAM, HR_adj_ 0.49 (95% CI 0.26–0.94; *P*_interaction_ = 0.032). *AHR*_7 AG was associated with increased risk for death in non-TAM-treated patients, but not in TAM-treated patients, using *AHR*_7 AA as reference (Supplementary Fig. 3E, F). There was also an interaction between the *AHR*_9 Any A genotype and use of sequential therapy on overall survival, HR_adj_ 2.72 (95% CI 1.18–6.25; *P*_interaction_ = 0.019). Among patients who were treated with TAM/AI or AI/TAM, a two-fold increased risk for death was seen in patients with the *AHR*_9 Any A genotype compared to patients with the *AHR*_9 GG genotype, but not in patients without sequential therapy (Supplementary Fig. 3G, H).

After further adjustment for the lifestyle factors that interacted with *AHR* (alcohol abstention, smoking and antioxidant intake), the effect sizes of the interactions with adjuvant treatments revealed essentially the same results. However, for AI-treated *AHR*_3 GA carriers, the interaction became borderline weaker (from *P*_interaction_ = 0.040 to *P*_interaction_ = 0.056). Taken together, *AHR* genotypes only seemed to impact prognosis in endocrine treated patients who had received AI alone or as sequential treatment.

### DGE analysis for *AHR* tertile 3 versus tertile 1

Since several SNPs were associated with *AHR* mRNA expression, the SCAN-B cohort was used for investigation of the differential impact of tumour *AHR* mRNA expression according to ER status. DGE analyses were performed tumours with high (T3) versus low (T1) *AHR* expression stratified by ER status. In ER+ tumours, 39 genes were upregulated, while two genes were downregulated, in *AHR*-high *vs. AHR*-low breast tumours (Supplementary Fig. 4; Supplementary Table 5). In *AHR*-high tumours, oestrogen responses, myogenesis and hypoxia hallmarks were enriched only in ER+ tumours (Supplementary Fig. 4C). In *AHR*-high ER- tumours, nine genes were upregulated and three genes were downregulated compared with *AHR*-low tumours (Supplementary Fig. 4; Supplementary Table 6). Hallmarks found only in ER- *AHR*-high *vs. AHR*-low tumours were interferon alpha (IFN-α) response, angiogenesis, peroxisome and fatty acid metabolism (Supplementary Fig. 4D). The main enriched hallmarks in *AHR*-high *vs. AHR*-low tumours found irrespective of ER status were related to immune response. In contrast, DNA repair pathways were enriched in *AHR*-low tumours.

## Discussion

The current study demonstrated significant interactions between *AHR* genotypes, lifestyle and systemic adjuvant treatments with respect to clinical outcomes in breast cancer. A total of eight *AHR* SNPs, one being functional, and eight *AHR* haplotypes were examined. The current study is an expansion of previous studies [8, 18].

In addition to genetic regulation, AhR is activated by different ligands [9–12, 26]. In the current study, the prognostic impact of different *AHR* genotypes was substantially modified by lifestyle and treatment exposures. The largest differences were seen for a combination of *AHR* minor alleles and smoking, alcohol, antioxidant supplements, chemotherapy or AIs. Surprisingly, the prognostic impact of *AHR* genotypes only differed in non-radiation therapy-treated patients.

To our knowledge, only few studies examined interactions between *AHR* genotypes and lifestyle in cancer. Two previous studies evaluated *AHR*_9 genotypes and smoking on lung cancer susceptibility, with contradictory results [39, 40]. We found no reports on *AHR* genotypes and smoking or alcohol in breast cancer. In the current study, there were no significant associations between *AHR*_9 and smoking or alcohol on prognosis. However, both exposures were associated with poorer prognosis in patients with *AHR*_1 Any T, linked to *AHR*_9. In a previous in vitro study, *AHR*_1 was found to regulate *AHR*-transcription in a polymorphism-dependent manner via the tumour suppressor nuclear factor 1-C (NF1C) in endometrial cancer [41]. NF1C can drive epithelial cell differentiation and/or apoptosis in mammary glands and may function as a tumour suppressor independent of ER status in breast cancer [42]. The *AHR*_1 T-allele is directly associated with lower *AHR* mRNA expression in whole blood. In the current study, exposure to smoking was associated with worse overall survival in patients with the major *AHR*_4 G-allele, associated with lower *AHR* mRNA expression.

We previously showed that breast cancer patients who used antioxidants preoperatively had higher frequency of nuclear AhR positive tumours compared to non-users, indicating AhR activation. In vitro studies then confirmed that antioxidants led to AhR activation with increased CYP1B1 levels in two breast cancer cell lines [26]. Helmig et al. reported that the *AHR*_9 A-allele conferred lower mRNA expression of *AHR*, *ARNT* and *CYP1B1* in white blood cells compared to the *AHR*_9 G-allele [43], suggesting lower activity for the A-allele. In the current study, there was no significant association between *AHR* genotypes and tumour-specific AhR protein levels in the cytoplasm or nuclei overall. However, patients with self-reported antioxidant use combined with the minor alleles of *AHR*_9 or *AHR*_6 conferring lower *AHR* expression, had poorer prognosis than other patients. These results suggest that genotypes with lower *AHR* activity combined with antioxidant supplements may be harmful for breast cancer patients. Moreover, antioxidants may interact with chemo- and radiation therapy, since these partly work through generation of reactive oxygen species [44].

Among patients with the *AHR*_11 Any C and/or CGATTAGC genotypes, chemotherapy was associated with a significantly increased risk for breast cancer events and death, while chemonaïve patients had somewhat decreased risk. In these genotypes there is a shift from A to C on *AHR*_11, located in the 3*'*-UTR downstream of the coding sequence, possibly modifying regulatory elements and downstream signalling pathways [45]. Since the results remained essentially the same after adjustment for antioxidants, adjuvant chemotherapy may be less beneficial and confer more side-effects in patients with these *AHR* genotypes. The role of *AHR* genotypes in chemotherapy merits further study.

Approximately two-thirds of the patients in this study received radiation therapy, which may be affected by antioxidants [26, 46] or smoking [23] through modification of free radicals or hypoxia levels [47]. In contrast to the other exposures, *AHR* genotypes were only associated with differential clinical outcomes in non-radiation therapy-treated patients. This was also true after further adjustments for antioxidant use, smoking and mastectomy as final type of surgery. In the DEG analysis in the SCAN-B cohort, *AHR*-high expression was associated with hypoxia in ER+ tumours only. AhR requires heterodimerization with ARNT for activation. ARNT is also a dimerization partner of the hypoxia inducible factor-1 alpha (HIF-1α) for hypoxia signalling and a coactivator of ER signalling. HIF-1α and ER would be highly active in *AHR*-high ER+ tumours [48]. Moreover, *AHR*-high expression was associated with inflammatory response, IL-2 and IL-6 signalling, and tumour necrosis factor alpha (TNF-α) signalling via NF-κB in both ER+ and ER- tumours. AhR has both pro- and anti-tumour regulatory effects depending on the context and immune cell subset. AhR activation is critical in macrophage polarisation and NK cell cytolytic activity [49]. The immune system is affected by both radiation therapy and chemotherapy [50], and we hypothesise that the impact on the immune system may be influenced by *AHR* genotypes. In addition, DGE analyses indicated that DNA repair pathways are enriched in *AHR* low expressing tumours corresponding to genotypes containing minor alleles (except for *AHR*_4), which might impact the efficacy of radiation and chemotherapy [51].

Among AI-treated patients with ER+ tumours, *AHR*_3 heterozygosity negatively impacted clinical outcome. Further, among non-TAM-treated patients, *AHR*_7 heterozygosity was associated with increased risk for death. These results may indicate that patients with ER+ tumours combined with *AHR*_3 GA may derive less benefit from AI treatment, while *AHR*_7 AG carriers may benefit from TAM treatment, compared to patients homozygous for the major alleles. In the current study, patients with the *AHR*_9 Any A genotype, combined with sequential therapy, had a two-fold increased risk for death, but not for breast cancer events, which is partly in line with previous results [18].

The BCblood cohort is a population-based observational cohort with reliable anthropometric and clinicopathological data, representative for its catchment area with long-term follow-up [23]. Questionnaires were completed preoperatively, which limits recall bias. However, alcohol, smoking and antioxidant supplement use were self-reported, which presents a risk for self-representational bias. Another limitation was that the majority of study participants were of European descent, although ethnicity was not recorded. Genotype frequencies and lifestyle differ between ethnic groups. The study is largely exploratory and the results therefore need validation in other cohorts, as well as in mechanistic studies. Over 90% of SNPs associated with clinical phenotypes are located in noncoding regions. To fully understand the functional implications of common SNPs, it is insufficient to study eQTLs only under basal conditions [52]. It is currently unknown how representative the evaluated SNPs in the current study are with respect to all SNPs affecting AhR regulation/activation after exposure to various compounds. The majority of SNPs on the OncoArray platform were intronic. Hence, *AHR* SNPs and haplotypes that capture a larger region were examined, but no further imputation of other potentially functional SNPs was performed. However, based on the results from the current study, the genotyped SNPs and haplotypes may still be useful as markers. Additional sequencing could provide information on rare, unlinked and functional variants, but performing validation of the function of such variants was outside the scope of this study. Due to low frequency of homozygous minor allele carriers, six SNPs and six haplotypes were dichotomized, which limits the ability to study recessive traits. The multivariable models were adjusted for known confounders to assess whether *AHR* combined with lifestyle or adjuvant treatments hold independent prognostic information. Nevertheless, residual confounding remains possible.

In conclusion, different *AHR* genotypes were found to significantly interact with lifestyle factors and adjuvant treatments with respect to breast cancer prognosis. The impact of *AHR* genotypes varied according to smoking status, alcohol consumption, antioxidant supplement use, chemotherapy, radiation therapy and endocrine treatment type. Exception for radiation therapy, poorer prognosis was seen in exposed patients carrying *AHR* genotypes linked to lower *AHR* mRNA expression, possibly due to the role of AhR as master regulator of metabolism, hypoxia, DNA repair and immune response. If confirmed, these results emphasise the importance of considering pharmacogenomics, including *AHR* genotypes, for more personalised lifestyle recommendations and to better guide selection of adjuvant breast cancer treatment.

## Supplementary information

Supplementary Material

## Data Availability

Data from the BCblood study are not publicly available due to privacy laws and questions regarding data should be directed at the corresponding author. Clinical and RNA-seq data from SCAN-B is accessible from Staaf et al. [32], available at Mendeley Data.
