# Supplementary material for: Interplay between AHR genotypes, lifestyle factors and adjuvant breast cancer treatments significantly impacts clinical outcome in a population-based cohort
Source: BJC Rep. 2025 Jul 11;3:51. doi: 10.1038/s44276-025-00167-w (PMC12254277; doi:10.1038/s44276-025-00167-w)
Supplement: Supplementary file 1 — Supplementary Material [file 44276_2025_167_MOESM1_ESM.pdf]

Interplay between *AHR* genotypes, lifestyle factors and adjuvant breast cancer treatments significantly impacts clinical outcome in a population-based cohort

Annelie Augustinsson[1], Christopher Godina[1], Linn Nilsson[1,2], Kelin Gonçalves de Oliveira[1], Karolin Isaksson[3,4] and Helena Jernström[1*]

[1]Department of Clinical Sciences in Lund, Oncology, Lund University, and Lund University Cancer Center LUCC/Kamprad, Lund, Sweden

[2]Department of Medical Physics and Engineering, Växjö Central Hospital and Department of Research and Development, Region Kronoberg, Växjö, Sweden

[3]Department of Clinical Sciences in Lund, Surgery, Lund University, and Lund University Cancer Center LUCC, Lund, Sweden

[4]Kristianstad Hospital, Kristianstad, Sweden

E-mail: helena.jernstrom@med.lu.se

**Table of contents**	**Page**
Supplementary Figure Legends	2
Supplementary Figure 1	3
Supplementary Figure 2	4
Supplementary Figure 3	5
Supplementary Figure 4	6
Supplementary Table 1	7
Supplementary Table 2	8
Supplementary Table 3	9–10
Supplementary Table 4	11–12
Supplementary Table 5	13
Supplementary Table 6	14

Supplementary Figure Legends

Supplementary Figure 1 Kaplan-Meier estimates of breast cancer-free interval in relation to *AHR*_1, with LogRank and HR_{adj} (95% CI), in (**A**) alcohol abstainers and (**B**) alcohol drinkers. Overall survival in relation to *AHR*_1, with LogRank and HR_{adj} (95% CI), in (**C**) alcohol abstainers and (**D**) alcohol drinkers. Overall survival in relation to CAGTTGGA genotype, with LogRank and HR_{adj} (95% CI), in (**E**) alcohol abstainers and (**F**) alcohol drinkers. The number of patients is indicated at each time-point. The study is ongoing. Thus, the number of patients decreases with time.

Supplementary Figure 2 Kaplan-Meier estimates of breast cancer-free interval in relation to CGGTTAGA or CGGCTAGA genotypes, with LogRank and HR_{adj} (95% CI), in (**A, C**) non-radiotherapy and (**B, D**) radiotherapy treated patients. The number of patients is indicated at each time-point. The study is ongoing. Thus, the number of patients decreases with time.

Supplementary Figure 3 Kaplan-Meier estimates of breast cancer-free interval in relation to *AHR*_3, with LogRank and HR_{adj} (95% CI) in (**A**) non-AI and (**B**) AI-treated patients. Overall survival in relation to *AHR*_3, with LogRank and HR_{adj} (95% CI), in (**C**) non-AI-treated and (**D**) AI-treated patients. Overall survival in relation to *AHR*_7, with LogRank and HR_{adj} (95% CI), in (**E**) non-TAM-treated and (**F**) TAM-treated patients. Overall survival in relation to *AHR*_9 genotypes, with LogRank and HR_{adj} (95% CI), in (**G**) non-sequential-treated and (**H**) sequential-treated patients. The number of patients is indicated at each time-point. The study is ongoing. Thus, the number of patients decreases with time.

Supplementary Figure 4 Molecular analyses of *AHR* expression. Volcano plot showing significant up- and downregulated genes in *AHR* high expressing (T3) compared to *AHR* low expressing (T1) tumours in (**A**) ER+ and (**B**) ER- tumours. Dot plots showing activated and suppressed Hallmark signatures in *AHR* T3 compared to *AHR* T1 in (**C**) ER+ and (**D**) ER- tumours. Hallmarks denoted in red were found irrespective of tumour ER status, whereas hallmarks denoted in black were exclusive to either ER+ or ER- tumours. Expression data for patients in SCAN-B were downloaded from Staaf *et al*. [1] and gene expression profiles from 5,326 unique patients with unilateral breast cancer and distant metastasis follow-up were used [2].

References

1. Staaf, J., et al., *RNA sequencing-based single sample predictors of molecular subtype and risk of recurrence for clinical assessment of early-stage breast cancer.* NPJ Breast Cancer, 2022. **8**(1): p. 94.).
2. Godina, C., et al., *Caveolin-1 gene expression provides additional prognostic information combined with PAM50 risk of recurrence (ROR) score in breast cancer.* Sci Rep, 2024. **14**(1): p. 6675.

Supplementary Figure 1

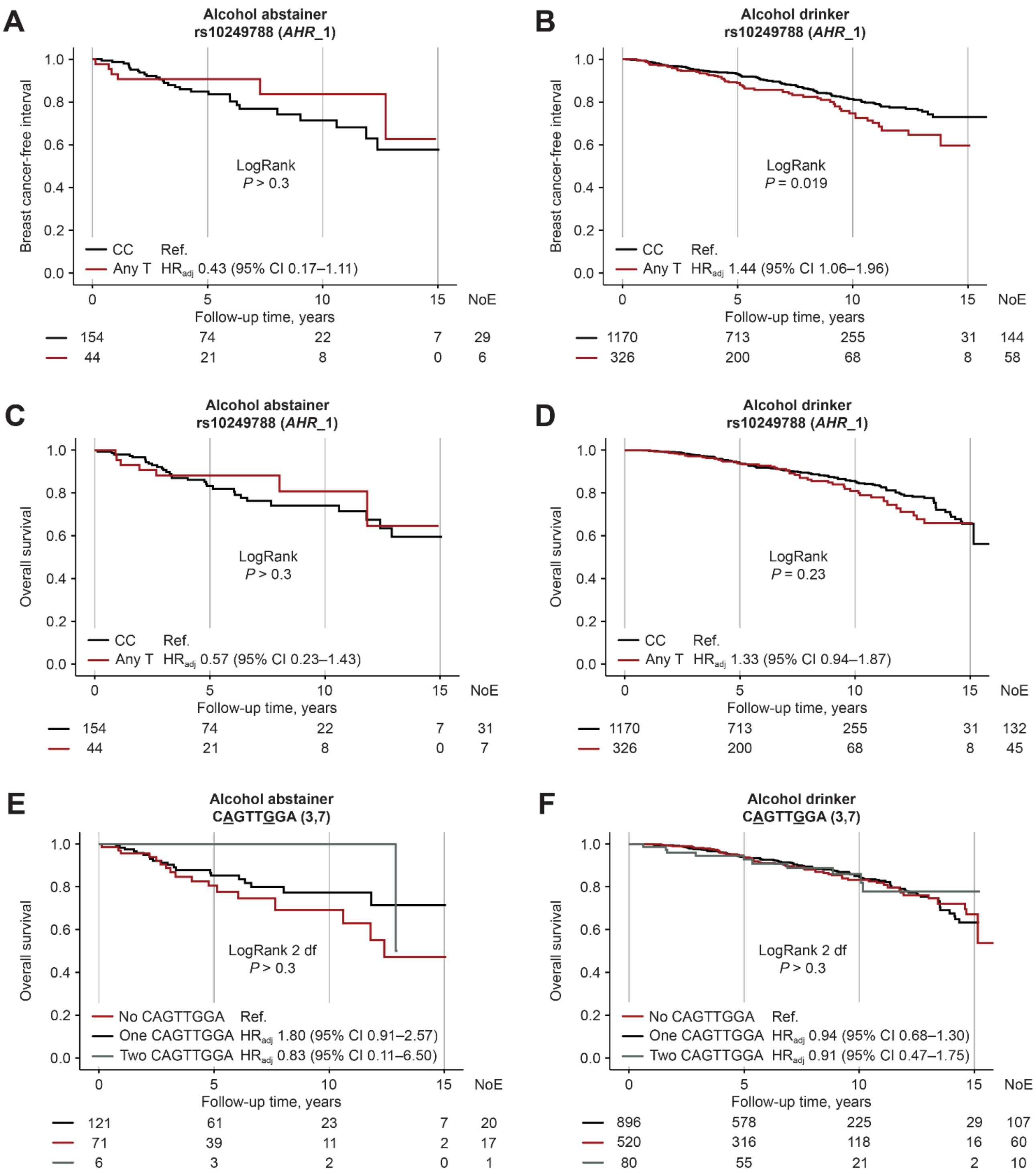

Supplementary Figure 2

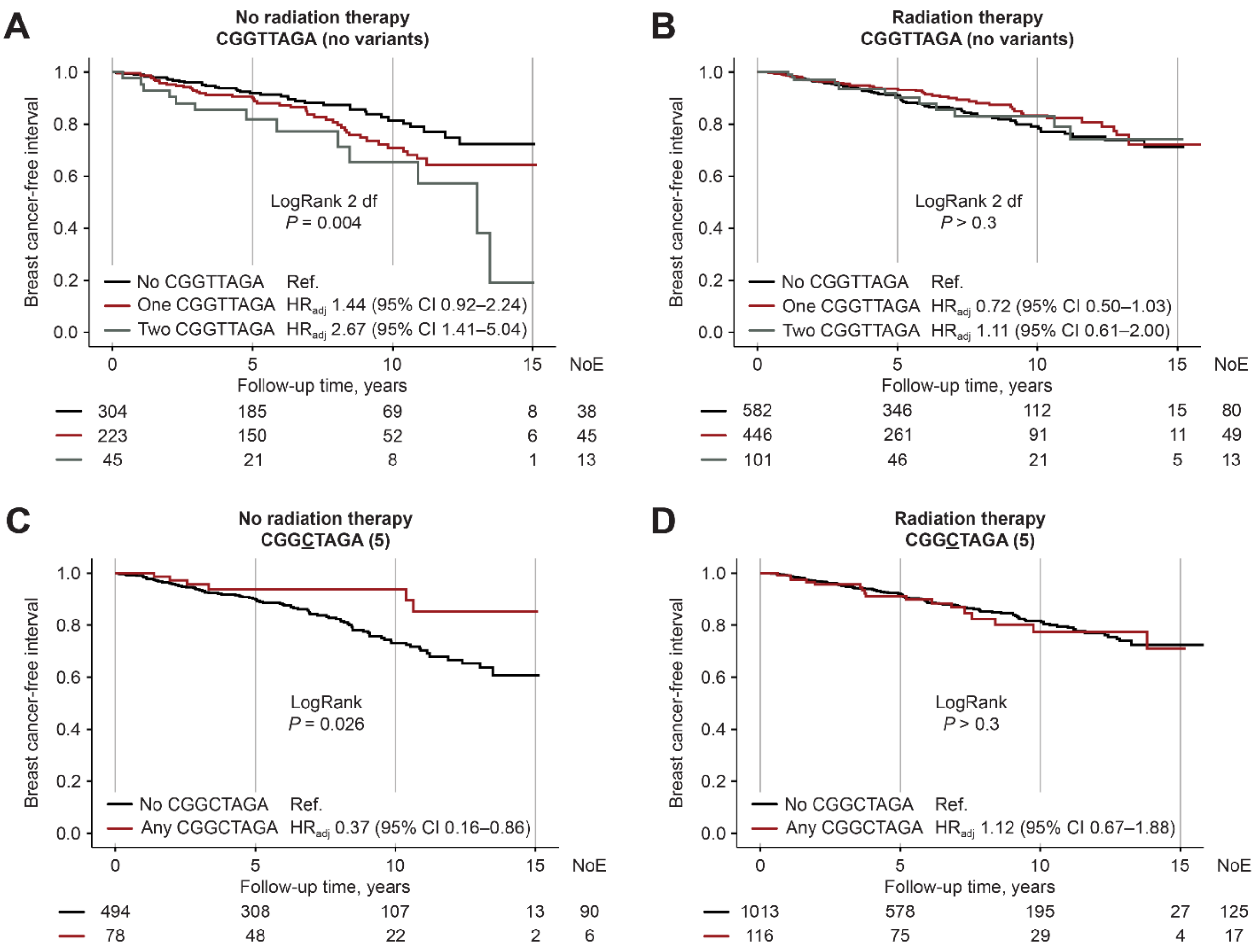

Supplementary Figure 3

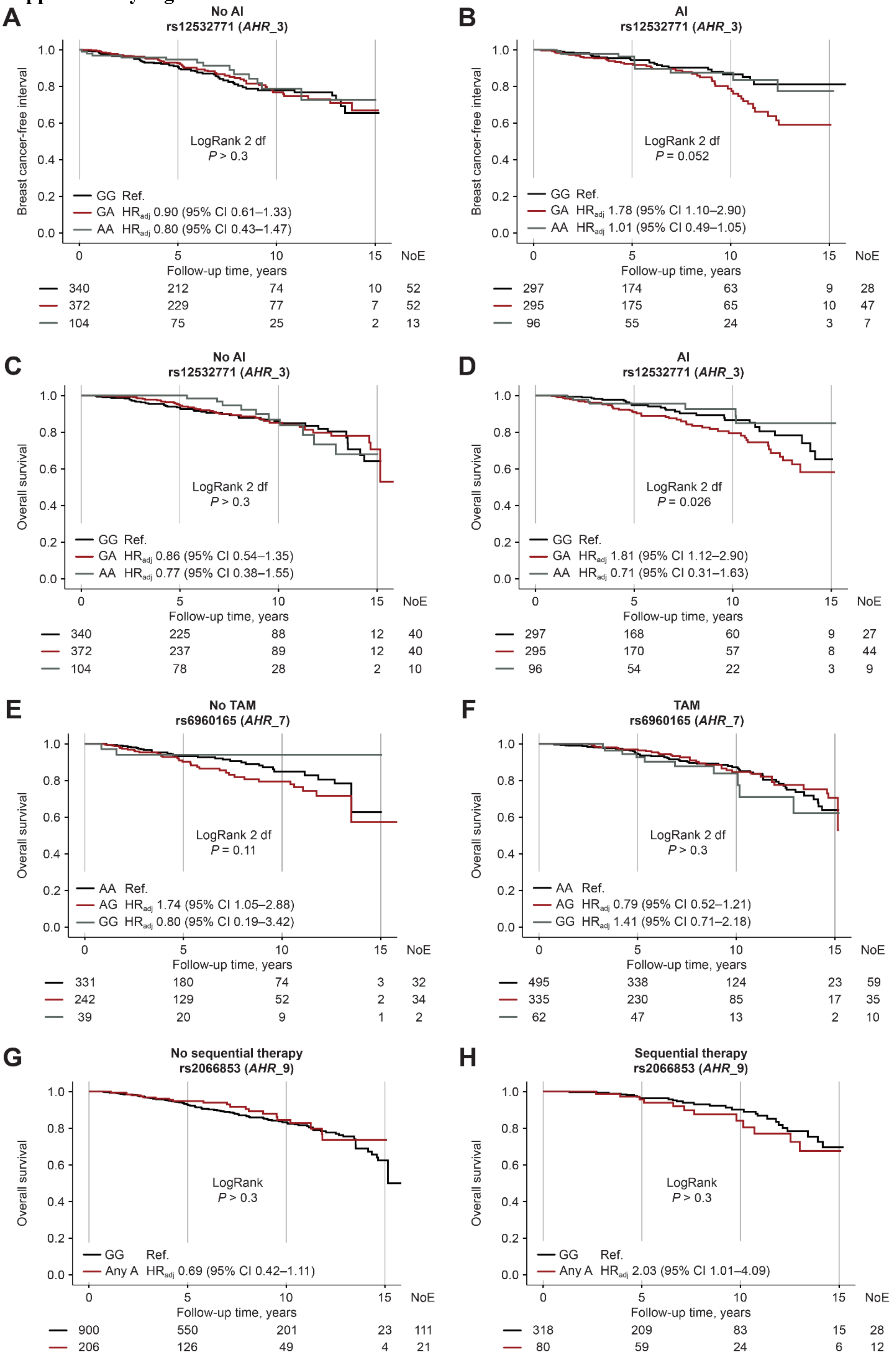

Supplementary Figure 4

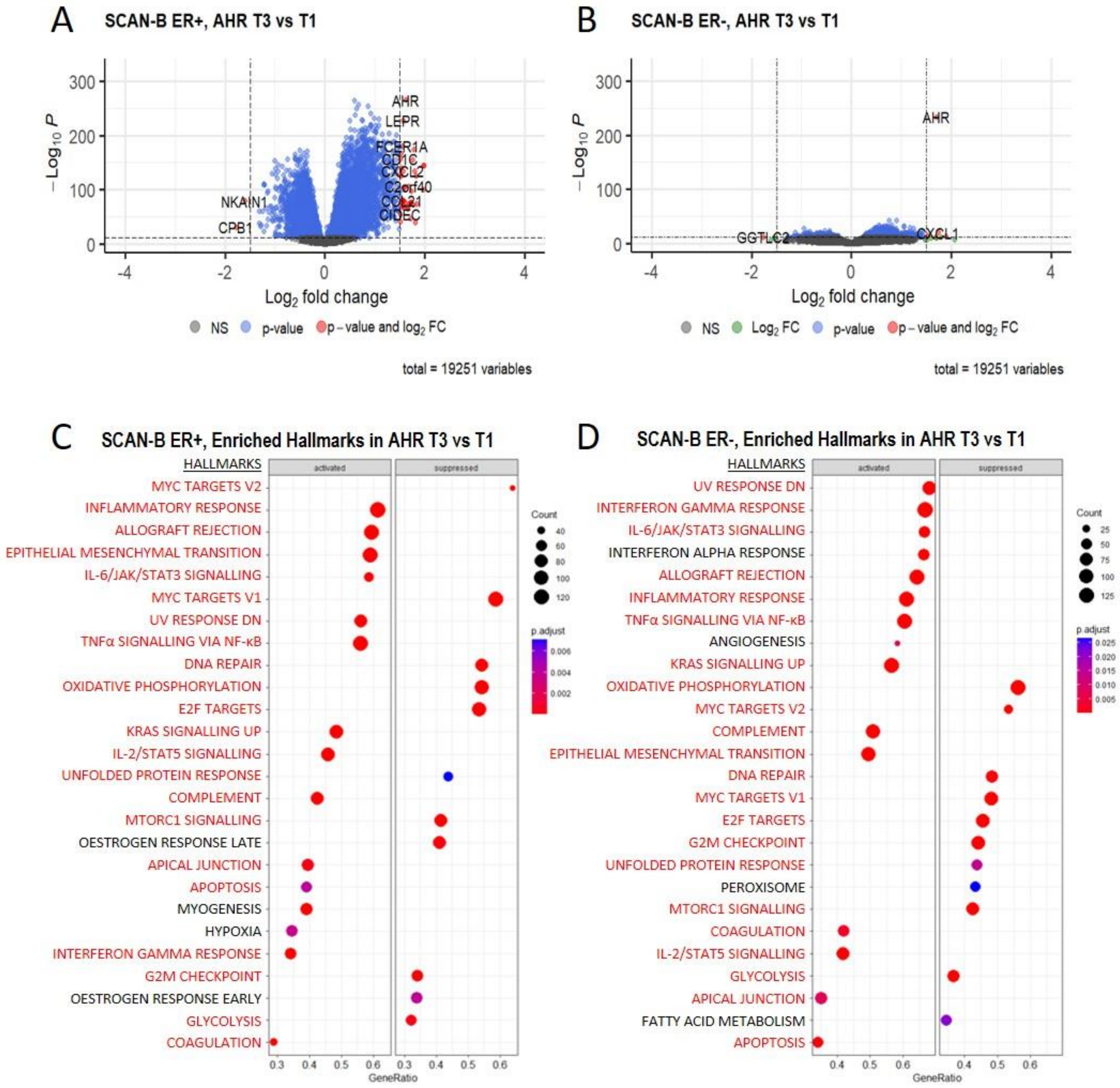

Supplementary Table 1 Characteristics of the 15 *AHR* SNPs on the OncoArray

AHR	rs-ID	Location*	Variant	Call rate	Included
1	rs10249788	7:17338147	2 KB upstream transcript/intron variant	1,701 (100.0%)	Yes
2	rs10253202	7:17338425	5'-UTR/upstream transcript/intron variant	Bad quality, failure rate >5%	No
3	rs12532771	7:17341796	Intron variant	1,701 (100.0%)	Yes
4	rs17722841	7:17343594	Intron variant	1,701 (100.0%)	Yes
5	rs17779352	7:17349626	Synonymous/coding sequence variant	1,701 (100.0%)	Yes
6	rs17137566	7:17360521	Intron variant	1,692 (99.5%)	Yes
7	rs6960165	7:17368085	Intron variant	1,701 (100.0%)	Yes
8	rs1176108462	7:17375302	Missense/coding sequence variant	Non-informative, SNP rate <1%	No
9	rs2066853	7:17379110	Missense/coding sequence variant	1,701 (100.0%)	Yes
10	rs34185983	7:17382938	3'-UTR variant	Non-informative, SNP rate <1%	No
11	rs4987097	7:17383061	3'-UTR variant	1,678 (98.6%)	Yes
12	rs17137616	7:17383865	3'-UTR variant	Non-informative, SNP rate <1%	No
13	rs35618592	7:17384210	3'-UTR variant	Non-informative, SNP rate <1%	No
14	rs35877514	7:17385247	3'-UTR variant	Non-informative, SNP rate <1%	No
15	rs115894490	7:17385682	3'-UTR variant	Non-informative, SNP rate <1%	No

*According to dbSNP, Assembly 37 (NCBI, NHI)

Supplementary Table 2 Predicted haplotypes in the European 1000Genome human population compared to the BCblood cohort

Haplotype	rs10249788 (*AHR*_1)	rs12532771 (*AHR*_3)	rs17722841 (*AHR*_4)	rs17779352 (*AHR*_5)	rs17137566 (*AHR*_6)	rs6960165 (*AHR*_7)	rs2066853 (*AHR*_9)	rs4987097 (*AHR*_11)	Frequency 1000Genome	BCblood
No. 1	C	G	G	T	T	A	G	A	0.3320	0.2825
No. 2	C	A	G	T	T	G	G	A	0.2028	0.2252
No. 3	C	G	A	T	T	A	G	A	0.1282	0.1449
No. 4	T	A	G	T	C	A	A	A	0.0984	0.0908
No. 5	C	G	G	T	C	A	G	A	0.0706	0.0641
No. 6	C	G	G	C	T	A	G	A	0.0567	0.0591
No. 7	T	A	G	T	T	A	G	A	0.0278	0.0247
No. 8	C	G	A	T	T	A	G	C	0.0258	0.0262
No. 9	C	G	G	T	T	G	G	A	0.0179	0.0359
No. 10	C	G	A	C	T	A	G	C	0.0129	0.0241
Rare <0.01									0.0269	0.0226

Supplementary Table 3 The impact of *AHR* SNPs on *AHR* mRNA expression (Gene code ID ENSG00000106546.12)

Query	RS ID	Position grch37	R2	D'	Gene	Tissue	Non effect allele freq	Effect allele freq	Effect size	*P*-value
rs10249788 (*AHR*_1)	rs2301677	chr7:17326058	0.538014350945858	1	*AHR*	Whole Blood	T=0.782	C=0.218	-0.10974	8.05363e-06
rs10249788 (*AHR*_1)	rs10249788 (*AHR*_1)	chr7:17338147	1	1	*AHR*	Whole Blood	C=0.87	T=0.13	-0.143121	7.85352e-07
rs10249788 (*AHR*_1)	rs2237297	chr7:17359594	0.671352599736886	0.93367032967033	*AHR*	Whole Blood	G=0.897	A=0.103	-0.141517	4.90832e-05
rs10249788 (*AHR*_1)	rs2074113	chr7:17373771	0.679478720688909	0.934302040816327	*AHR*	Whole Blood	G=0.896	T=0.104	-0.142239	4.9006e-05
rs10249788 (*AHR*_1)	rs2066853 (*AHR*_9)	chr7:17379110	0.664866783129431	0.905069462647444	*AHR*	Whole Blood	G=0.892	A=0.108	-0.158904	8.43133e-07
rs10249788 (*AHR*_1)	rs202198518	chr7:17384574	0.671761327636148	0.924075471698113	*AHR*	Whole Blood	G=0.895	-=0.105	-0.144326	3.54162e-05
rs10249788 (*AHR*_1)	rs10238167	chr7:17389885	0.625285359926293	0.891536388140162	*AHR*	Whole Blood	G=0.895	A=0.105	-0.161486	5.62099e-07
rs10249788 (*AHR*_1)	rs142598791	chr7:17390260	0.631885841842231	0.910701803051318	*AHR*	Whole Blood	CT=0.898	-=0.102	-0.135863	9.9682e-05
rs10249788 (*AHR*_1)	rs138179132	chr7:17392620	0.839717823923784	0.920404395604396	*AHR*	Whole Blood	C=0.871	A=0.129	-0.124727	2.58617e-05
rs10249788 (*AHR*_1)	rs10269143	chr7:17398983	0.333818390395113	0.701771288816423	*AHR*	Whole Blood	T=0.819	A=0.181	-0.108164	4.05588e-05
rs10249788 (*AHR*_1)	rs7791070	chr7:17401027	0.474014743487385	0.941378707534526	*AHR*	Whole Blood	T=0.781	C=0.219	-0.113881	4.19691e-06
rs10249788 (*AHR*_1)	rs1993247	chr7:17403102	0.617266106212037	0.890503401360544	*AHR*	Whole Blood	A=0.896	G=0.104	-0.156278	8.0876e-07
rs10249788 (*AHR*_1)	rs10274243	chr7:17403696	0.593325119009301	0.887282913165266	*AHR*	Whole Blood	G=0.899	A=0.101	-0.145565	8.23378e-06
rs10249788 (*AHR*_1)	rs7780687	chr7:17405718	0.2996221546119	0.680417091014417	*AHR*	Whole Blood	A=0.812	G=0.188	-0.108017	6.6127e-05
rs10249788 (*AHR*_1)	rs1111544	chr7:17414784	0.286362594439274	0.654334057688379	*AHR*	Whole Blood	A=0.817	G=0.183	-0.108129	8.88288e-05
rs10249788 (*AHR*_1)	rs2123744	chr7:17417300	0.286362594439274	0.654334057688379	*AHR*	Whole Blood	G=0.817	T=0.183	-0.11389	4.34857e-05
rs12532771 (*AHR*_3)	rs10249788 (*AHR*_1)	chr7:17338147	0.274076005304375	0.988293613852169	*AHR*	Whole Blood	C=0.87	T=0.13	-0.143121	7.85352e-07
rs12532771 (*AHR*_3)	rs2237297	chr7:17359594	0.197407368248943	0.955763367729831	*AHR*	Whole Blood	G=0.897	A=0.103	-0.141517	4.90832e-05
rs12532771 (*AHR*_3)	rs2074113	chr7:17373771	0.199702662657332	0.956184668989547	*AHR*	Whole Blood	G=0.896	T=0.104	-0.142239	4.9006e-05
rs12532771 (*AHR*_3)	rs2066853 (*AHR*_9)	chr7:17379110	0.202842672162722	0.943723428059969	*AHR*	Whole Blood	G=0.892	A=0.108	-0.158904	8.43133e-07
rs12532771 (*AHR*_3)	rs202198518	chr7:17384574	0.202003133879722	0.956598021168891	*AHR*	Whole Blood	G=0.895	-=0.105	-0.144326	3.54162e-05
rs12532771 (*AHR*_3)	rs10238167	chr7:17389885	0.189967788113865	0.927663368614818	*AHR*	Whole Blood	G=0.895	A=0.105	-0.161486	5.62099e-07
rs12532771 (*AHR*_3)	rs142598791	chr7:17390260	0.189082893817589	0.940445181150841	*AHR*	Whole Blood	CT=0.898	-=0.102	-0.135863	9.9682e-05
rs12532771 (*AHR*_3)	rs138179132	chr7:17392620	0.252517532361836	0.95281425891182	*AHR*	Whole Blood	C=0.871	A=0.129	-0.124727	2.58617e-05
rs12532771 (*AHR*_3)	rs1993247	chr7:17403102	0.187687707388524	0.926974448315912	*AHR*	Whole Blood	A=0.896	G=0.104	-0.156278	8.0876e-07
rs12532771 (*AHR*_3)	rs10274243	chr7:17403696	0.186807832133501	0.93986131037781	*AHR*	Whole Blood	G=0.899	A=0.101	-0.145565	8.23378e-06
rs12532771 (*AHR*_3)	rs12699845	chr7:17443286	0.22377561547643	0.845468509984639	*AHR*	Testis	C=0.63	T=0.37	0.141232	1.74775e-05
rs12532771 (*AHR*_3)	rs7794392	chr7:17450853	0.232632579225108	0.867580332946187	*AHR*	Testis	C=0.633	T=0.367	0.16041	1.11045e-06
rs12532771 (*AHR*_3)	rs56985649	chr7:17452873	0.167670899738335	0.45666087869488	*AHR*	Testis	CATAAGGAAA=0.3	-=0.7	0.135356	7.02915e-06
rs12532771 (*AHR*_3)	rs12699848	chr7:17459230	0.303766008180579	0.750062111801242	*AHR*	Testis	C=0.497	T=0.503	0.127434	1.92878e-05
rs17722841 (*AHR*_4)	rs7794392	chr7:17450853	0.222213387930047	0.795622864530428	*AHR*	Testis	C=0.633	T=0.367	0.16041	1.11045e-06
rs17137566 (*AHR*_6)	rs2301677	chr7:17326058	0.681049436051188	0.935729344870912	*AHR*	Whole Blood	T=0.782	C=0.218	-0.10974	8.05363e-06
rs17137566 (*AHR*_6)	rs10249788 (*AHR*_1)	chr7:17338147	0.35078840519961	0.712138973757811	*AHR*	Whole Blood	C=0.87	T=0.13	-0.143121	7.85352e-07
rs17137566 (*AHR*_6)	rs2237297	chr7:17359594	0.53269580943651	1	*AHR*	Whole Blood	G=0.897	A=0.103	-0.141517	4.90832e-05
rs17137566 (*AHR*_6)	rs2074113	chr7:17373771	0.53841479671873	1	*AHR*	Whole Blood	G=0.896	T=0.104	-0.142239	4.9006e-05
rs17137566 (*AHR*_6)	rs2066853 (*AHR*_9)	chr7:17379110	0.512413395960901	0.955359817179371	*AHR*	Whole Blood	G=0.892	A=0.108	-0.158904	8.43133e-07
rs17137566 (*AHR*_6)	rs202198518	chr7:17384574	0.531729017699762	0.988524103944697	*AHR*	Whole Blood	G=0.895	-=0.105	-0.144326	3.54162e-05
rs17137566 (*AHR*_6)	rs10238167	chr7:17389885	0.483492357453699	0.942620519723483	*AHR*	Whole Blood	G=0.895	A=0.105	-0.161486	5.62099e-07
rs17137566 (*AHR*_6)	rs142598791	chr7:17390260	0.502388215726045	0.976379709090055	*AHR*	Whole Blood	CT=0.898	-=0.102	-0.135863	9.9682e-05
rs17137566 (*AHR*_6)	rs138179132	chr7:17392620	0.364012908379719	0.728639196353828	*AHR*	Whole Blood	C=0.871	A=0.129	-0.124727	2.58617e-05
rs17137566 (*AHR*_6)	rs10269143	chr7:17398983	0.927207988316312	0.972717904214352	*AHR*	Whole Blood	T=0.819	A=0.181	-0.108164	4.05588e-05
rs17137566 (*AHR*_6)	rs7791070	chr7:17401027	0.729697101989246	0.971398922484257	*AHR*	Whole Blood	T=0.781	C=0.219	-0.113881	4.19691e-06
rs17137566 (*AHR*_6)	rs1993247	chr7:17403102	0.477845023444663	0.942074048482755	*AHR*	Whole Blood	A=0.896	G=0.104	-0.156278	8.0876e-07
rs17137566 (*AHR*_6)	rs10274243	chr7:17403696	0.472746575051357	0.952296275221092	*AHR*	Whole Blood	G=0.899	A=0.101	-0.145565	8.23378e-06

rs17137566 (*AHR*_6)	rs7780687	chr7:17405718	0.872382525527093	0.965605191359586	*AHR*	Whole Blood	A=0.812	G=0.188	-0.108017	6.6127e-05
rs17137566 (*AHR*_6)	rs1111544	chr7:17414784	0.851605656053133	0.938465929943319	*AHR*	Whole Blood	A=0.817	G=0.183	-0.108129	8.88288e-05
rs17137566 (*AHR*_6)	rs2123744	chr7:17417300	0.851605656053133	0.938465929943319	*AHR*	Whole Blood	G=0.817	T=0.183	-0.11389	4.34857e-05
rs6960165 (*AH*R_9)	rs1077773	chr7:17442679	0.30151929972948	0.877110071080097	*AHR*	Testis	G=0.426	A=0.574	0.135597	2.7522e-06
rs6960165 (*AH*R_9)	rs12699844	chr7:17443199	0.297432530026294	0.87646778922163	*AHR*	Testis	C=0.429	T=0.571	0.134282	3.37731e-06
rs6960165 (*AH*R_9)	rs56985649	chr7:17452873	0.446945659447672	0.811148378053664	*AHR*	Testis	CATAAGGAAA=0.3	-=0.7	0.135356	7.02915e-06
rs6960165 (*AH*R_9)	rs4721601	chr7:17458078	0.386628179808069	0.781308542675013	*AHR*	Testis	C=0.315	A=0.685	0.142241	1.84071e-06
rs6960165 (*AH*R_9)	rs12699848	chr7:17459230	0.15650131334696	0.72849158120179	*AHR*	Testis	C=0.497	T=0.503	0.127434	1.92878e-05
rs6960165 (*AH*R_9)	rs12699849	chr7:17460989	0.410478349470026	0.818052630899014	*AHR*	Testis	C=0.322	T=0.678	0.138231	4.07961e-06
rs2066853 (*AH*R_9)	rs2301677	chr7:17326058	0.386972000644058	0.941363673455114	*AHR*	Whole Blood	T=0.782	C=0.218	-0.10974	8.05363e-06
rs2066853 (*AH*R_9)	rs10249788 (*AH*R_1)	chr7:17338147	0.664866783129431	0.905069462647444	*AHR*	Whole Blood	C=0.87	T=0.13	-0.143121	7.85352e-07
rs2066853 (*AH*R_9)	rs2237297	chr7:17359594	0.948839480054517	1	*AHR*	Whole Blood	G=0.897	A=0.103	-0.141517	4.90832e-05
rs2066853 (*AH*R_9)	rs2074113	chr7:17373771	0.959026158498712	1	*AHR*	Whole Blood	G=0.896	T=0.104	-0.142239	4.9006e-05
rs2066853 (*AH*R_9)	rs2066853 (*AH*R_9)	chr7:17379110	1	1	*AHR*	Whole Blood	G=0.892	A=0.108	-0.158904	8.43133e-07
rs2066853 (*AH*R_9)	rs202198518	chr7:17384574	0.969235474006116	1	*AHR*	Whole Blood	G=0.895	-=0.105	-0.144326	3.54162e-05
rs2066853 (*AH*R_9)	rs10238167	chr7:17389885	0.948834289737271	0.989419658820807	*AHR*	Whole Blood	G=0.895	A=0.105	-0.161486	5.62099e-07
rs2066853 (*AH*R_9)	rs142598791	chr7:17390260	0.918345106906591	0.989111493543743	*AHR*	Whole Blood	CT=0.898	-=0.102	-0.135863	9.9682e-05
rs2066853 (*AH*R_9)	rs138179132	chr7:17392620	0.702507088210974	0.926249423987265	*AHR*	Whole Blood	C=0.871	A=0.129	-0.124727	2.58617e-05
rs2066853 (*AH*R_9)	rs10269143	chr7:17398983	0.53790576574541	0.988799323060479	*AHR*	Whole Blood	T=0.819	A=0.181	-0.108164	4.05588e-05
rs2066853 (*AH*R_9)	rs7791070	chr7:17401027	0.42400837457488	0.988257814506151	*AHR*	Whole Blood	T=0.781	C=0.219	-0.113881	4.19691e-06
rs2066853 (*AH*R_9)	rs1993247	chr7:17403102	0.938648649237557	0.98931889366672	*AHR*	Whole Blood	A=0.896	G=0.104	-0.156278	8.0876e-07
rs2066853 (*AH*R_9)	rs10274243	chr7:17403696	0.908227056386079	0.989004743480447	*AHR*	Whole Blood	G=0.899	A=0.101	-0.145565	8.23378e-06
rs2066853 (*AH*R_9)	rs7780687	chr7:17405718	0.49028377844372	0.966110069284583	*AHR*	Whole Blood	A=0.812	G=0.188	-0.108017	6.6127e-05
rs2066853 (*AH*R_9)	rs1111544	chr7:17414784	0.44971704590956	0.910176566441215	*AHR*	Whole Blood	A=0.817	G=0.183	-0.108129	8.88288e-05
rs2066853 (*AH*R_9)	rs2123744	chr7:17417300	0.472181611917264	0.932632424830911	*AHR*	Whole Blood	G=0.817	T=0.183	-0.11389	4.34857e-05

Abbreviation: *AHR*, aryl hydrocarbon receptor

Supplementary Table 4 Patient characteristics at inclusion, tumour characteristics and adjuvant treatments in relation to *AHR* genotypes

			rs10249788 (*AHR*_1)		rs12532771 (*AHR*_3)			rs17722841 (*AHR*_4)		rs17779352 (*AHR*_5)	
	All patients	Missing	CC	Any T	GG	GA	AA	GG	Any A	TT	Any C
	n=1,701		*n*=1,330 (78.2%)	*n*=371 (21.8%)	*n*=716 (42.1%)	*n*=755 (44.4%)	*n*=230 (13.5%)	*n*=1,092 (64.2%)	*n*=609 (35.8%)	*n*=1,407 (82.7%)	*n*=294 (17.3%)
	n (%)	*n*	*n* (%)	*n* (%)	*n* (%)	n (%)	*n* (%)	*n* (%)	*n* (%)	*n* (%)	*n* (%)
Age ≥50 years	1,367 (80.4)	0	1,080 (81.2)	287 (77.4)	579 (80.9)	597 (79.1)	191 (83.0)	867 (79.4)	500 (82.1)	1,135 (80.7)	232 (78.9)
BMI ≥25 kg/m^2	836 (52.3)	101	651 (52.5)	185 (51.4)	349 (52.2)	364 (51.0)	123 (56.7)	541 (52.5)	295 (51.8)	692 (52.4)	144 (51.6)
Parous	1,509 (88.7)	0	1,175 (88.3)	334 (90.0)	628 (87.7)	672 (89.0)	209 (90.9)	979 (89.7)	530 (87.0)	1,242 (88.3)	267 (90.8)
Ever use of MHT	671 (39.6)	6	519 (39.1)	152 (41.3)	284 (39.7)	283 (37.7)	104 (45.2)	440 (40.4)	231 (38.1)	558 (39.8)	113 (38.4)
Preoperative smoker	300 (17.7)	7	245 (18.5)	55 (14.9)	134 (18.8)	123 (16.4)	43 (18.8)	200 (18.4)	100 (16.5)	237 (16.9)	63 (21.5)
Alcohol abstainer	198 (11.7)	7	154 (11.6)	44 (11.9)	88 (12.3)	81 (10.8)	29 (12.6)	136 (12.5)	62 (10.2)	160 (11.4)	38 (13.0)
Antioxidant supplement user	175 (10.4)	24	138 (10.5)	37 (10.2)	70 (9.9)	87 (11.7)	18 (7.9)	117 (10.9)	58 (9.6)	141 (10.2)	34 (11.8)
Invasive tumour size >20 mm or skin/muscular involvement	441 (25.9)	0	330 (24.8)	111 (29.9)	190 (26.5)	184 (24.4)	67 (29.1)	286 (26.2)	155 (25.5)	359 (25.5)	82 (27.9)
Axillary node involvement ≥1	579 (34.1)	2	443 (33.3)	136 (36.8)	251 (35.1)	251 (33.3)	77 (33.5)	375 (34.4)	204 (33.6)	474 (33.7)	105 (35.7)
Histological grade III	470 (27.7)	6	370 (27.9)	100 (27.0)	192 (26.9)	216 (28.8)	62 (27.0)	300 (27.6)	170 (28.0)	385 (27.5)	85 (28.9)
Hormone receptor status											
ER positive	1,504 (88.5)	2	1,173 (88.3)	331 (89.2)	637 (89.0)	667 (88.6)	200 (87.0)	961 (88.1)	543 (89.3)	1,244 (88.5)	260 (88.4)
PgR positive	1,214 (71.5)	2	947 (71.3)	267 (72.0)	509 (71.1)	539 (71.6)	166 (72.2)	788 (72.2)	426 (70.1)	1,006 (71.6)	208 (70.7)
HER2 amplification[a]	150 (10.7)	11	115 (10.5)	35 (11.4)	63 (10.7)	70 (11.2)	17 (9.1)	93 (10.4)	57 (11.1)	123 (10.6)	27 (11.3)
Triple negative	129 (7.6)	8	102 (7.7)	27 (7.3)	49 (6.9)	57 (7.6)	23 (10.0)	86 (7.9)	43 (7.1)	110 (7.9)	19 (6.5)
AhR localisation[b]											
Cytoplasmatic high	736 (80.1)	782	562 (79.6)	174 (81.7)	320 (80.4)	318 (81.3)	98 (75.4)	468 (80.4)	268 (79.5)	603 (79.7)	133 (82.1)
Nuclear positive	291 (31.7)	782	231 (32.7)	60 (28.2)	135 (33.9)	123 (31.5)	33 (25.4)	184 (31.6)	107 (31.8)	238 (31.4)	53 (32.7)
Treatment by last follow-up prior to any event											
Ever use of chemotherapy	488 (28.7)	0	375 (28.2)	113 (30.5)	199 (27.8)	221 (29.3)	68 (29.6)	307 (28.1)	181 (29.7)	409 (29.1)	79 (26.9)
Ever use of radiation therapy	1,129 (66.4)	0	878 (66.0)	251 (67.7)	468 (65.4)	493 (65.3)	168 (73.0)	728 (66.7)	401 (65.8)	940 (66.8)	189 (64.3)
Ever use of trastuzumab[a]	118 (8.3)	0	91 (8.2)	27 (8.7)	53 (8.9)	51 (8.1)	14 (7.4)	70 (7.8)	48 (9.3)	96 (8.2)	22 (9.2)
Patients with ER+ tumours, *n*=1,504											
Ever use of TAM	892 (59.3)	0	698 (59.5)	194 (58.6)	386 (60.6)	386 (57.9)	120 (60.0)	572 (59.5)	320 (58.9)	743 (59.7)	149 (57.3)
Ever use of AI	688 (45.7)	0	532 (45.4)	156 (47.1)	297 (46.6)	295 (44.2)	96 (48.0)	432 (45.0)	256 (47.1)	568 (45.7)	120 (46.2)
Ever use of endocrine treatment											
No	322 (21.4)	0	249 (21.2)	73 (22.1)	134 (21.0)	148 (22.2)	40 (20.0)	214 (22.3)	108 (19.9)	260 (20.9)	62 (23.8)
TAM only	494 (32.8)	0	392 (33.4)	102 (30.8)	206 (32.3)	224 (33.6)	64 (32.0)	315 (32.8)	179 (33.0)	416 (33.4)	78 (30.0)
AI only	290 (19.3)	0	226 (19.3)	64 (19.3)	117 (18.4)	133 (19.9)	40 (20.0)	175 (18.2)	115 (21.2)	241 (19.4)	49 (18.8)
Sequential therapy (TAM/AI or AI/TAM)	398 (26.5)	0	306 (26.1)	92 (27.8)	180 (28.3)	162 (24.3)	56 (28.0)	257 (26.7)	141 (26.0)	327 (26.3)	71 (27.3)

Abbreviations: AhR, aryl hydrocarbon receptor; AI, aromatase inhibitor; BMI, body mass index; ER, oestrogen receptor; HER2, human epidermal growth factor receptor 2; MHT, menopausal hormone therapy; PgR, progesterone receptor; TAM, tamoxifen

[a]2005–2016, *n*=1,415

[b]Adjusted for time between surgery and staining

Supplementary Table 4 Cont.

	rs17137566 (*AHR*_6)		rs6960165 (*AHR*_7)			rs2066853 (*AHR*_9)		rs4987097 (*AHR*_11)	
	TT	Any C	AA	AG	GG	GG	Any A	AA	Any C
	n=1,188 (69.8%)	*n*=513 (30.2%)	*n*=932 (54.8%)	*n*=656 (38.6%)	*n*=113 (6.6%)	*n*=1,379 (81.1%)	*n*=322 (18.9%)	*n*=1,506 (88.5%)	*n*=195 (11.5%)
	n (%)	*n* (%)	*n* (%)	n (%)	*n* (%)	*n* (%)	*n* (%)	*n* (%)	*n* (%)
Age ≥50 years	959 (80.7)	408 (79.5)	740 (79.4)	533 (81.3)	94 (83.2)	1,115 (80.9)	252 (78.3)	1,209 (80.3)	158 (81.0)
BMI ≥25 kg/m^2	567 (51.1)	269 (54.8)	468 (53.1)	311 (50.7)	57 (54.3)	671 (52.1)	165 (53.1)	741 (52.4)	95 (51.4)
Parous	1,046 (88.0)	463 (90.3)	817 (87.7)	592 (90.2)	100 (88.5)	1,216 (88.2)	293 (91.0)	1,337 (88.8)	172 (88,2)
Ever use of MHT	475 (40.1)	196 (38.4)	362 (39.0)	258 (39.4)	51 (45.1)	540 (39.3)	131 (40.9)	597 (39.8)	74 (37.9)
Preoperative smoker	222 (18.8)	78 (15.3)	162 (17.4)	113 (17.3)	25 (22.1)	251 (18.3)	49 (15.3)	268 (17.9)	32 (16.4)
Alcohol abstainer	135 (11.4)	63 (12.3)	114 (12.3)	76 (11.6)	8 (7.1)	154 (11.2)	44 (13.7)	182 (12.1)	16 (8.3)
Antioxidant supplement user	123 (10.5)	52 (10.3)	97 (10.6)	69 (10.6)	9 (8.0)	144 (10.6)	31 (9.8)	158 (10.6)	17 (8.8)
Invasive tumour size >20 mm or skin/muscular involvement	299 (25.2)	142 (27.7)	252 (27.0)	165 (25.2)	24 (21.2)	351 (25.5)	90 (28.0)	381 (25.3)	60 (30.8)
Axillary node involvement ≥1	400 (33.7)	179 (35.0)	336 (36.1)	210 (32.1)	33 (29.2)	462 (33.5)	117 (36.4)	500 (33.2)	79 (40.5)
Histological grade III	332 (28.0)	138 (27.0)	255 (27.4)	186 (28.5)	29 (25.7)	380 (27.7)	90 (28.0)	413 (27.5)	57 (29.2)
Hormone receptor status									
ER positive	1,045 (88.0)	459 (89.6)	826 (88.6)	577 (88.2)	101 (89.4)	1,218 (88.5)	286 (88.8)	1,324 (88.0)	180 (92.3)
PgR positive	838 (70.6)	376 (73.4)	668 (71.7)	462 (70.6)	84 (74.3)	980 (71.2)	234 (72.7)	1,077 (71.6)	137 (70.3)
HER2 amplification[a]	104 (10.5)	46 (11.0)	88 (11.4)	57 (10.5)	5 (5.5)	121 (10.6)	29 (11.2)	132 (10.6)	18 (11.0)
Triple negative	92 (7.8)	37 (7.2)	65 (7.0)	54 (8.3)	10 (8.8)	104 (7.6)	25 (7.8)	121 (8.1)	8 (4.1)
AhR localisation[b]									
Cytoplasmatic high	507 (79.8)	229 (80.6)	424 (81.2)	263 (78.7)	49 (77.8)	588 (80.0)	148 (80.4)	645 (80.0)	91 (80.5)
Nuclear positive	205 (32.3)	86 (30.3)	170 (32.6)	101 (30.2)	20 (31.7)	238 (32.4)	53 (28.8)	259 (32.1)	32 (28.3)
Treatment by last follow-up prior to any event									
Ever use of chemotherapy	343 (28.9)	145 (28.3)	271 (29.1)	189 (28.8)	28 (24.8)	392 (28.4)	96 (29.8)	428 (28.4)	60 (30.8)
Ever use of radiation therapy	786 (66.2)	343 (66.9)	616 (66.1)	428 (65.2)	85 (75.2)	909 (65.9)	220 (68.3)	998 (66.3)	131 (67.2)
Ever use of trastuzumab[a]	81 (8.2)	37 (8.7)	76 (9.8)	37 (6.7)	5 (5.5)	94 (8.1)	24 (9.2)	102 (8.1)	16 (9.8)
Patients with ER+ tumours, *n*=1,504									
Ever use of TAM	610 (58.4)	282 (61.4)	495 (59.9)	335 (58.1)	62 (61.4)	727 (59.7)	165 (57.7)	784 (59.2)	108 (60.0)
Ever use of AI	474 (45.4)	214 (46.6)	384 (46.5)	262 (45.4)	42 (41.6)	546 (44.8)	142 (49.7)	603 (45.5)	85 (47.2)
Ever use of endocrine treatment									
No	231 (22.1)	91 (19.8)	174 (21.1)	127 (22.0)	21 (20.8)	263 (21.6)	59 (20.6)	292 (22.1)	30 (16.7)
TAM only	340 (32.5)	154 (33.6)	268 (32.4)	188 (32.6)	38 (37.6)	409 (33.6)	85 (29.7)	429 (32.4)	65 (36.1)
AI only	204 (19.5)	86 (18.7)	157 (19.0)	115 (19.9)	18 (17.8)	228 (18.7)	62 (21.7)	248 (18.7)	42 (23.3)
Sequential therapy (TAM/AI or AI/TAM)	270 (25.8)	128 (27.9)	227 (27.5)	147 (25.5)	24 (23.8)	318 (26.1)	80 (28.0)	355 (26.8)	43 (23.9)

Abbreviations: AhR, aryl hydrocarbon receptor; AI, aromatase inhibitor; BMI, body mass index; ER, oestrogen receptor; HER2, human epidermal growth factor receptor 2; MHT, menopausal hormone therapy; PgR, progesterone receptor; TAM, tamoxifen

[a]2005–2016, *n*=1,415

[b]Adjusted for time between surgery and staining

Supplementary Table 5 List of up- and downregulated genes in ER+ tumours in relation to *AHR* high expressing (T3) compared to *AHR* low expressing (T1) tumours

Upregulated = FDR adj. *P*-value≤0.05 & $log_2FC \geq 1.5$

Upregulated genes (HGNC) (*n*=39)	Entrez GeneID	Log_2FC	AveExpr	adj.*P*.Value
AHR	196	1.621192277	4.34608167	0
LEPR	3953	1.569678342	2.2973398	1.596E-228
FCER1A	2205	1.557265894	2.5801524	3.533E-181
ABCA8	10351	1.774673008	1.54275294	1.684E-174
ANGPTL1	9068	1.525776632	1.60952854	4.565E-168
CD1C	911	1.516064683	1.14789004	7.392E-158
OGN	4969	1.751944805	3.95116808	2.703E-156
C7	730	1.983269377	2.92827388	1.786E-145
SFRP1	6422	1.96993682	3.5870087	4.681E-145
MMRN1	22915	1.517480698	1.48365743	1.61E-140
CHRDL1	91851	1.790912616	3.05609713	1.079E-135
CXCL2	2920	1.570362169	0.12643172	2.571E-135
OSR1	130497	1.5017303	0.52751004	3.302E-127
SCARA5	286133	1.822418925	0.38367525	1.105E-126
LYVE1	10894	1.522245279	1.44236057	1.985E-126
C2ORF40	84417	1.66836913	1.22541775	2.588E-107
ADH1C	126	1.576174132	0.67011333	5.357E-105
RERGL	79785	1.571770438	0.62752139	2.339E-104
ADH1B	125	1.968909796	3.53655881	2.635E-101
PROM1	8842	1.722159509	1.56475706	6.5003E-98
CCL21	6366	1.557500695	2.72870475	2.9301E-81
COL17A1	1308	1.548045292	1.76778065	1.1843E-80
SOX10	6663	1.57494214	-0.2600377	1.5079E-80
GABRP	2568	1.529015088	1.51763341	1.0517E-77
PI16	221476	1.601270963	1.33875325	1.1744E-77
FABP4	2167	1.711198042	5.38437812	2.806E-77
STAC2	342667	1.728244057	0.29079776	3.254E-77
TFAP2B	7021	1.831234973	1.80781691	3.3287E-74
KRTS	3852	1.69287849	3.27982892	6.6185E-74
FDCSP	260436	1.766304876	1.24642706	4.832E-72
OPRPN	58503	1.560000213	-1.2046471	6.8795E-72
SLC34A2	10568	1.519138262	0.46013719	3.6771E-69
ADIPOQ	9370	1.640004935	2.57159339	1.2641E-68
VTCN1	79679	1.569800523	4.7858499	1.3352E-68
KRT14	3861	1.65555009	3.53405522	2.6536E-65
CIDEC	63924	1.51474317	1.76909484	3.3813E-55
CYP4Z1	199974	1.715625727	3.09321086	1.839E-47
OBP2B	29989	1.507869402	0.00410085	2.3176E-41
SCGB2A2	4250	1.805461501	7.66328265	1.5074E-40

Downregulated = FDR adj. *P*-value≤0.05 & $log_2FC \leq -1.5$

Downregulated genes (HGNC) (*n*=2)	Entrez GeneID	Log_2FC	AveExpr	adj.*P*.Value
NKAIN1	79570	-1.605699591	2.23795891	3.6572E-80
CPB1	1360	-1.777775502	3.53207284	5.4304E-32

Supplementary Table 6 List of up- and downregulated genes in ER- tumours in relation to *AHR* high expressing (T3) compared to *AHR* low expressing (T1) tumours

Upregulated = FDR adj. *P*-value≤0.05 & $log_2FC \geq 1.5$

Upregulated genes (HGNC) (*n*=9)	Entrez GeneID	Log_2FC	AveExpr	adj.*P*.Value
AHR	196	1.69389305	4.26131336	6.828E-235
CXCL1	2919	1.74175784	0.95978933	1.8445E-20
SCARA5	286133	1.5201302	-0.1052607	1.6937E-14
		1.89245221	0.83759867	4.0132E-13
C7	730	1.68735129	1.54623841	1.5203E-12
PI3	5266	1.79828901	1.53603255	2.3766E-11
CAPN6	827	1.59028268	1.0132059	8.8638E-11
SLC34A2	10568	1.69971263	2.40479564	3.9428E-10
KRT6B	3854	1.50359986	3.07099502	1.0921E-07
FDCSP	260436	2.06174145	3.88636999	3.301E-07

Downregulated = FDR adj. *P*-value≤0.05 & $log_2FC \leq -1.5$

Downregulated genes (HGNC) (*n*=3)	Entrez GeneID	Log_2FC	AveExpr	adj.*P*.Value
GGTLC2	91227	-1.7590555	-0.7564787	1.5209E-12
CLPSL1	340204	-1.5506089	1.07009587	2.7017E-11
ABCC11	85320	-1.6124646	0.4718674	6.2346E-06
